# Vanillin formation from ferulic acid in *Vanilla planifolia* is catalysed by a single enzyme

**DOI:** 10.1038/ncomms5037

**Published:** 2014-06-19

**Authors:** Nethaji J. Gallage, Esben H. Hansen, Rubini Kannangara, Carl Erik Olsen, Mohammed Saddik Motawia, Kirsten Jørgensen, Inger Holme, Kim Hebelstrup, Michel Grisoni, Birger Lindberg Møller

**Affiliations:** 1 Plant Biochemistry Laboratory, Department of Plant and Environmental Sciences, Faculty of Science, University of Copenhagen, Thorvaldsensvej 40, Frederiksberg C, DK-1871 Copenhagen, Denmark; 2 VILLUM Research Center ‘Plant Plasticity’, Thorvaldsensvej 40, Frederiksberg C, DK-1871 Copenhagen, Denmark; 3 Center for Synthetic Biology: ‘bioSYNergy’, Thorvaldsensvej 40, Frederiksberg C, DK-1871 Copenhagen, Denmark; 4 Evolva A/S, Lersø Parkallé 42–44, 5th floor, DK-2100 Copenhagen, Denmark; 5 AU Flakkebjerg, Danish Centre for Food and Agriculture, University of Aarhus, Forsøgsvej, DK-4200 Slagelse, Denmark; 6 Centre de Coopération Internationale en Recherche Agronomique pour le Dévelopement, UMR PVBMT, 97410 Saint Pierre, La Réunion, France; 7 Carlsberg Laboratory, Gamle Carlsberg Vej 10, Valby DK-2500, Copenhagen, Denmark

## Abstract

Vanillin is a popular and valuable flavour compound. It is the key constituent of the natural vanilla flavour obtained from cured vanilla pods. Here we show that a single hydratase/lyase type enzyme designated vanillin synthase (*Vp*VAN) catalyses direct conversion of ferulic acid and its glucoside into vanillin and its glucoside, respectively. The enzyme shows high sequence similarity to cysteine proteinases and is specific to the substitution pattern at the aromatic ring and does not metabolize caffeic acid and *p*-coumaric acid as demonstrated by coupled transcription/translation assays. *Vp*VAN localizes to the inner part of the vanilla pod and high transcript levels are found in single cells located a few cell layers from the inner epidermis. Transient expression of *VpVAN* in tobacco and stable expression in barley in combination with the action of endogenous alcohol dehydrogenases and UDP-glucosyltransferases result in vanillyl alcohol glucoside formation from endogenous ferulic acid. A gene encoding an enzyme showing 71% sequence identity to *Vp*VAN was identified in another vanillin-producing plant species *Glechoma hederacea* and was also shown to be a vanillin synthase as demonstrated by transient expression in tobacco.

## Abstract (web summary)

Vanilla is derived from vanillin isolated from a vanillin-producing orchid, but the process is laborious, costly and results in a small yield. Here, the authors identified an enzyme from the orchid, *Vanilla planifolia*, that is able to catalyse the formation of vanillin and vanillin glucoside from ferulic acid and its glucoside *in vitro*, respectively.

## Graphical abstract

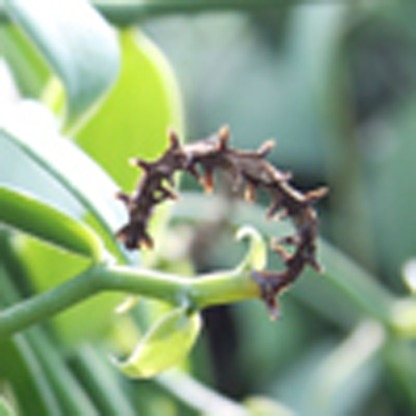

Vanilla is the world’s most popular flavour principle and used in numerous products. The pods of the climbing orchids, *Vanilla planifolia* and *V. tahitensis* are the source of natural vanilla^1^, although trace amounts of vanillin can be found in a variety of different plant species scattered in the plant kingdom^2^. Vanillin (3-methoxy-4-hydroxybenzaldehyde) is the main flavour component of vanilla extract from cured vanilla pods^1^,^3^. In high concentrations vanillin is toxic to living cells. In the pod it is produced and stored as non-toxic vanillin glucoside, which upon tissue damage is hydrolysed to form the active defense compound, vanillin. Production of vanillin from the orchids is laborious, slow and costly. Five hundred kilograms of vanilla pods yields only 1 kg of vanillin. Less than 1% of the global vanillin production originates from the vanilla orchids. Instead, the vast majority is produced chemically from fossil fuels or by acid hydrolysis of lignin^4^. A biotechnological solution to vanillin production via heterologous expression of the native vanilla orchid pathway genes in microorganisms has not been possible because the pathway has remained unknown. Vanillin has been produced by microbial bioconversion of substrates structurally related to vanillin^5^ as well as from glucose^6^.

Previous studies have shown the conversion of a variety of compounds into vanillin glucoside after administration to *V. planifolia* pods. These studies suggest that vanillin glucoside is derived from phenylalanine, the shikimate pathway intermediates or monomeric lignin precursors^7^,^8^,^9^,^10^,^11^,^12^. Vanillin glucoside and *p*-hydroxybenzaldehyde glucoside, the two most abundant aroma compounds in mature vanilla pods, are structurally similar, and a biosynthetic relationship between the formation of these two compounds has been proposed^12^,^13^. The necessary chain shortening process of a putative phenylpropanoid precursor was suggested to proceed by β-oxidation or by a coenzyme A (CoA)-dependent non-β-oxidative pathway^11^,^14^,^15^,^16^,^17^. *p*-Hydroxybenzaldehyde was reported to be formed by chain shortening of *p*-coumaric acid catalysed by *p*-hydroxybenzaldehyde synthase (4-HBS)^18^,^19^, which was proposed as a precursor for vanillin glucoside biosynthesis. *p*-Hydroxybenzaldehyde would then need to be hydroxylated at its C3 carbon by a monooxygenase (C3H), *O*-methylated at the 3-OH position by a *O*-methyltransferase (OMT), and finally glucosylated at the 4-OH position by a UDP-glucosyltransferase (UGT).

Other studies have suggested that vanillin is formed from L-phenylalanine via the monomeric lignin precursors: cinnamic acid, *p*-coumaric acid, caffeic acid and ferulic acid, involving phenylalanine ammonia lyase (PAL), hydroxylations, an *O*-methylation and finally a chain-shortening reaction. Formation of vanillin glucoside would also require the involvement of a UGT, although the point at which the glycosylation would take place remains elusive. Enzymes are known that can catalyse most of these reactions, for example, PAL^20^, cinnamic acid 4-hydroxylase, C4H^21^, *p*-coumaric acid 3-hydroxylase, C3H^22^, but is not clear whether CoA-derivatives are involved or whether the C3-hydroxylation step proceeds, for example, through quinate- and shikimate esters^22^. Caffeic acid could in principle be *O*-methylated^23^ to form ferulic acid, the substrate for the hypothesized final chain-shortening reaction, and several OMTs are known from *V. planifolia*. Vanillin UGTs or genes that encode these enzymes from *V. planifolia* have not yet been reported and as stated above it remains to be demonstrated at which stage in the pathway glycosylation occurs.

The aim of the current study was to elucidate the vanillin biosynthesis pathway in *V. planifolia*. We have carried out biosynthetic studies with fresh vanilla pods using a number of different putative radiolabelled precursors. Supported by a combination of transcriptomic and proteomic approaches, we identified a gene, *VpVAN* encoding a two-carbon chain-shortening enzyme converting ferulic acid and its glucoside directly into vanillin and its glucoside. *Vp*VAN is produced in cells in the inner part of the vanilla pod. The substrate specificity of *Vp*VAN was determined using a rabbit reticulocyte-coupled *in vitro* transcription/translation system and by heterologous expression of the gene in *Nicotiana benthamiana*, *Hordeum vulgare* and *Saccharomyces cerevisiae*. A gene sequence identical to that of *VpVAN* was previously reported to encode an enzyme designated 4-HBS catalysing a two-carbon chain-shortening of *p*-coumaric acid into 4-hydroxybenzaldehyde. We could not verify such a function in our studies.

## Results

### Administration of putative [^14^C]- precursors to vanilla pods

**Figure 1:**
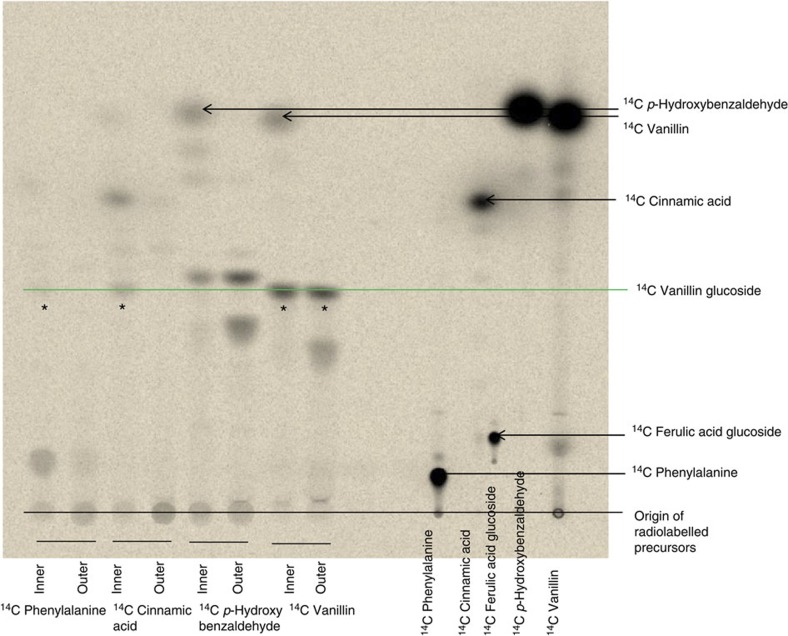
Thin layer chromatography (TLC) analysis of the radiolabelled products formed following administration of different putative ^14^C-labelled precursors to tissue slices of fresh vanilla pods. The ^14^C-labelled products formed following 48 h incubation were extracted into 70% MeOH, separated by TLC and monitored by autoradiography. The position of the radiolabelled precursors following chromatographic separation is shown on the right part of the thin layer. (*) indicates formation of ^14^C-labelled vanillin glucoside from the administered precursor. The presence of ultraviolet-absorbing components in the vanilla extracts as well as the position of the putative substrates after chromatographic separation was also monitored following exposure to ultraviolet light (254 nm) (Supplementary Fig. 1).

To examine whether the *p*-hydroxybenzaldehyde-based or the longer lignin precursor-based pathway is the most likely native vanillin glucoside biosynthetic pathway, [^14^C]-radiolabelled putative precursors ([^14^C]-phenylalanine, [^14^C]-cinnamic acid, [^14^C]-*p*-hydroxybenzaldehyde and [^14^C]-vanillin) were administered to sliced discs of fresh vanilla pods harvested 6 months after pollination. Vanillin glucoside is located in the inner part of the pod, that is, in the papillae and placental tissues, but completely absent from the epicarp, outer mesocarp area and seeds^24^. Accordingly, the experiments with administration of radiolabelled precursors were carried out separately with inner and outer parts of the pod discs using the outer parts of the pod as negative controls. Incubation with [^14^C]-phenylalanine and [^14^C]-cinnamic acid resulted in [^14^C]-vanillin glucoside formation in the tissue representing the inner part of the pod while administration of [^14^C]-*p*-hydroxybenzaldehyde induced [^14^C]-*p*-hydroxybenzaldehyde glucoside formation in both the inner and the outer part of the pod (Fig. 1; Supplementary Fig. 1). Incubation with [^14^C]-*p*-hydroxybenzaldehyde did not result in [^14^C]-vanillin glucoside formation. The radiolabelling studies confirmed that vanillin glucoside biosynthesis occurs only in the inner part of the pod and demonstrated that *p*-hydroxybenzaldehyde is not an intermediate in vanillin biosynthesis. The incorporation percentages observed varied depending upon the pod developmental stage, whereas the pattern of radiolabelled compounds observed following administration of each of the different labelled precursors at different developmental stages remained similar.

Administration of [^14^C]-vanillin resulted in formation of [^14^C]-vanillin glucoside both in the inner and outer part of the pod. Similarly [^14^C] *p*-hydroxybenzaldehyde administration resulted in [^14^C]-*p*-hydroxybenzaldehyde glucoside both in the inner and outer part of the pod, demonstrating the presence of a glycosyltransferase capable of glycosylating these precursors.

### Identification of candidate genes

To identify genes and enzymes involved in vanillin glucoside biosynthesis in *V. planifolia*, a combination of transcriptomic and proteomic approaches was undertaken with an initial focus on candidates representing the five major enzyme families suggested from the literature to play a possible role in vanillin biosynthesis, namely PAL, cytochrome P450s (the monophenol monooxygenases C4H and C3H), OMTs, UGTs and the carbon chain-shortening enzyme, 4-HBS. The *V. planifolia* transcriptome was obtained from a 6-month-old vanilla pod from the island of La Réunion by 454 pyrosequencing. Approximately 40 *UGTs*, 15 *OMTs*, a *CYP*98A3 (C3H) and *4-HBS* conreads were found in the transcriptome. To further assess the likelihood of involvement of each of these genes in vanillin biosynthesis, a targeted proteomic approach (proteomic mass finger printing) was carried out in parallel with the broad transcriptome analysis using the biosynthetically active inner part of the pod as experimental tissue. On the basis of overlay of the pyrosequencing and proteomic data sets, we selected and cloned 1UGT (*VpUGT72U1*), 11OMTs, a *CYP98A3* orthologue (*Vp*C*YP98A70*) and *4-HBS* (Supplementary Table 1; Supplementary Data 1 and 2).

Although in the literature, the vanillin biosynthetic pathway has been suggested to be embedded within a metabolic grid, our initial *in vitro* studies with these gene candidates identified a gene encoding an enzyme converting ferulic acid glucoside and ferulic acid directly into vanillin glucoside and vanillin, respectively. This represents the first committed step in vanillin synthesis and demonstrates that vanillin formation in *V. planifolia* is catalysed by a single enzyme using a general substrate from phenylpropanoid metabolism. We designated the enzyme vanillin synthase and the gene *VpVAN* (gene sequence is given in Supplementary Fig. 2a). In a published patent application, the identical gene sequence had previously been assigned as encoding an enzyme converting *p*-coumaric acid into *p*-hydroxybenzaldehyde^18^. Accordingly the gene sequence was initially designated as encoding a *p-*hydroxybenzaldehyde synthase (4-HBS), an activity that we did not observe in our studies as reported below.

### Vanillin synthase catalyses vanillin formation *in vitro*

**Figure 2:**
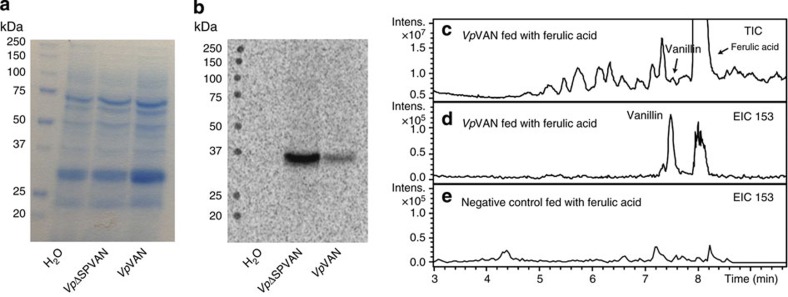
Direct coupled transcription/translation of the PCR-generated DNA for *Vp*VAN and for *Vp*VAN devoid of its 21 amino-acid-long ER-targeting signal peptide (*Vp*ΔSPVAN). L-[^35^S]-methionine was included to specifically monitor the formation of *de novo* synthesized radiolabelled proteins by SDS–PAGE. The ability of *Vp*VAN synthesized by *in vitro* transcription/translation to catalyse conversion of ferulic acid into vanillin was monitored by LC–MS using total and selected ion monitoring. (**a**) Proteins present in the *in vitro* transcription/translation assay visualized by Coomassie brilliant blue staining. (**b**) The [^35^S]-labelled *Vp*ΔSPVAN and *Vp*VAN proteins formed from the two PCR products as visualized by autoradiography. In each transcription/translation experiment, a single radiolabelled protein band of the expected approximate mass was obtained. (**c**) Incubation of the transcription translation protein solution containing *Vp*VAN with 5 mM of ferulic acid for 1 h in 2.5 mM dithiothreitol at 30 °C, total ion chromatogram (TIC) following LC–MS analysis; (**d**) EIC: 153: extracted ion chromatogram for *m*/*z* vanillin (M+H^+^) for specific detection of vanillin formation. (**e**) EIC 153: extracted ion chromatogram for *m*/*z* vanillin (M+H^+^) of a control experiment in which ferulic acid was administered to a transcription translation protein solution not expressing *Vp*VAN. Intens., intensity.

The catalytic activity of vanillin synthase using a range of different putative substrates was monitored by *in vitro* coupled transcription/translation assays. Vanillin synthase protein was obtained from its PCR-generated DNA in a coupled transcription/translation assay with the inclusion of L-[^35^S]-methionine to provide easy monitoring of protein formation by SDS–polyacrylamide gel electrophoresis (PAGE) analysis (Fig. 2). The coupled assay produced a single radiolabelled protein band migrating with an apparent molecular mass of 36 kD in close agreement with the predicted mass of 39.15 kD for *Vp*VAN (Fig. 2), thus enabling us to monitor the activity of the enzyme in an *in vitro* condition equalling a purified enzyme.

**Figure 3:**
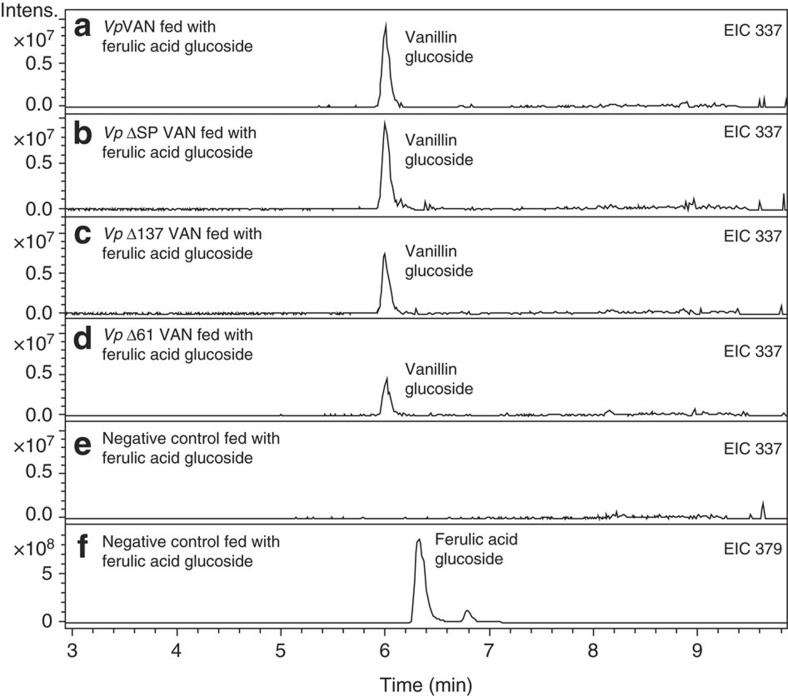
The ability of enzymes synthesized by *in vitro* transcription/translation to catalyse conversion of ferulic acid glucoside into vanillin glucoside. The experiment shown in (**a**–**d**) involved incubation of the transcription/translation protein solutions with *Vp*VAN, *Vp*ΔSPVAN, *Vp*Δ137VAN and *Vp*Δ61VAN with 1 mM of ferulic acid glucoside for 1 h in 1 mM dithiothreitol at 30 °C. (**a**) EIC 337: extracted ion chromatogram *m*/*z* vanillin glucoside (M+Na^+^) obtained following incubation of *Vp*VAN with ferulic acid glucoside (**b**) EIC 337: extracted ion chromatogram *m*/*z* vanillin glucoside (M+Na^+^) obtained following incubation of *Vp*ΔSPVAN with ferulic acid glucoside (**c**) EIC 337: extracted ion chromatogram *m*/*z* vanillin glucoside (M+Na^+^) obtained following incubation of *Vp*Δ137VAN with ferulic acid glucoside. (**d**) EIC 337: extracted ion chromatogram *m*/*z* vanillin glucoside (M+ Na^+^) obtained following incubation of *Vp*Δ61VAN with ferulic acid glucoside. (**e**) EIC 337: extracted ion chromatogram *m*/*z* vanillin glucoside (M+Na^+^) obtained following incubation of a control transcription translation/ protein solution devoid of any protein of interest with ferulic acid glucoside. (**f**) EIC 379: extracted ion chromatogram *m*/*z* ferulic acid glucoside (M+Na^+^) obtained following incubation of a control transcription translation/ protein solution devoid of any protein of interest with ferulic acid glucoside. Intens., intensity.

The substrate specificity of the *Vp*VAN enzyme formed was investigated by incubation (1 h and 24 h) with 0.5–5 mM of *p*-coumaric acid, caffeic acid, ferulic acid, *p*-coumaric acid glucoside, caffeic acid glucoside, ferulic acid glucoside, feruloyl-CoA, *p*-coumaroyl-CoA and caffeoyl-CoA. Liquid chromatography–mass spectrometry (LC–MS) analyses demonstrated that *Vp*VAN catalysed a direct chain shortening of ferulic acid and ferulic acid glucoside to vanillin and vanillin glucoside, respectively (Figs 2 and 3), whereas no activity was found using *p*-coumaric acid, caffeic acid and the glucosides of these as substrates. The conversion of ferulic acid and ferulic acid glucoside into vanillin and vanillin glucoside proceeded in the absence of added adenosine tri-phosphate (ATP) and nicotinamide adenine dinucleotide (NAD^+^). These cofactors would have been required if the chain shortening had occurred via β-oxidation of an activated CoA ester^16^. In a number of experiments, vanillin formation was observed using feruloyl-CoA as a substrate. In such experiments, the incubation mixture was found always to contain ferulic acid present as an impurity in the commercially provided feruloyl-CoA. Since ATP and NAD^+^ were not required as cofactors in these reactions, we conclude that vanillin synthase is not able to utilize feruloyl-CoA as substrate for vanillin formation by β-oxidation of the activated CoA ester^16^. Accordingly, we conclude that *Vp*VAN is catalysing vanillin and vanillin glucoside formation from ferulic acid and ferulic acid glucoside in a coupled non-oxidative hydratase/lyase reaction.

A general amino-acid sequence identity search using GenBank showed that the *Vp*VAN protein exhibits high sequence identity to cysteine proteinases. The highest amino-acid sequence identity (77%) was found to the *Elaeis guineensis* cysteine proteinase belonging to the aleurain class of cysteine proteinases (MEROPS-the peptidase database). Interestingly, alignments unequivocally demonstrated that the *Vp*VAN sequence contained the three key active site residues required for proteinase activity^25^. Likewise the *Vp*VAN amino-acid sequence contains the typical processing motif known from cysteine proteinases, indicating that the *Vp*VAN protein when produced in the vanilla pod may be subject to a maturation cycle involving cleavage after the amino-acid residue at position 137 (DGV/LPVT). To test whether the activity of the *Vp*VAN enzyme would be altered in the absence of the pro-peptide or if it changes the catalytic activity of the enzyme *in vitro*, we generated two truncated versions of *Vp*VAN. The first truncated version was designed to lack the first 137 amino acids (*vp*Δ137*van).* A second truncated version of *Vp*VAN was designed lacking the first 61amino acids (*vp*Δ61*van*) to reflect processing at a second possible peptide cleavage site predicted by the ProP 1.0 Server. Hydropathy plot analysis indicated that the *Vp*VAN sequence also contains an N-terminal endoplasmic reticulum (ER)-targeting signal peptide (first 21 amino-acid residues), and a truncated *Vp*VAN with no ER-targeting signal peptide (*vp*Δ*spvan*) was therefore also constructed and tested *in vitro* using the coupled transcription/translation assay. Neither the presence of the target sequence nor the presence of the pro-peptide has a significant influence on the activity of the *Vp*VAN enzyme (Fig. 3).

### Cellular localization of vanillin synthase in *V. planifolia*

**Figure 4:**
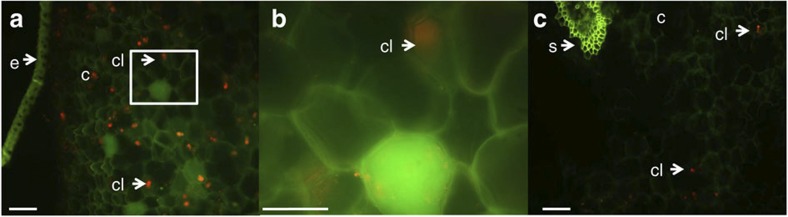
Tissue localization of the expression of *Vp*VAN in transverse sections of a 6-month-old vanilla pod determined by in tube *in situ* PCR. Transcripts of *VpVAN* were detected in specific cells (Panels **a** and **b**) using FITC-conjugated antibodies recognizing digoxigenin (DIG) incorporated in the specific PCR products representing the *VpVAN* transcript. Higher magnification of the selected area in Panel **a** is shown in Panel **b**. No transcripts of *VpVAN* were detected in the controls in the absence of specific primers for *VpVAN* (Panel **c**). The fluorescence detected in Panel **c** represents unspecific binding of the FITC-conjugated antibodies recognizing DIG to cell walls and supporting fibre cells surrounding the vascular systems. In all panels the chloroplasts are visible owing to their auto fluorescence at the used filter settings. The sections were analysed with a Leica FI/RH filter with excitation filters: BP490/15; 560/25 and emission filters: BP525/20; 605/30. e, epidermis; c, cortex; cl, chloroplast; s, supporting fibre tissue; Scale bar, 100 μm.

The cellular localization of transcripts encoding vanillin synthase in the vanilla orchid was determined. The proteome analyses showed that the *Vp*VAN enzyme is localized in the inner part of the pod, the tissue actively synthesizing vanillin glucoside. In tube *in situ* PCR^26^ was used to determine the cellular localization of transcripts encoding *Vp*VAN (Fig. 4). The analyses were performed on tissue sections from vanilla pod discs using primers specific to *VpVAN*. Six-month-old pods were selected based on their high biosynthetic activity compared with younger pods. *VpVAN* transcripts were detected in the segment of the pod discs representing the inner part of the vanilla pod. High transcript levels were observed in single cells located a few cell layers from the epidermis facing the inner cavity of the pod (Fig. 4), supplementing the conclusion from the biosynthetic and proteomic studies that vanillin glucoside biosynthesis occurs in the inner part of the pod.

### The catalytic activity of *Vp*VAN in yeast

**Figure 5:**
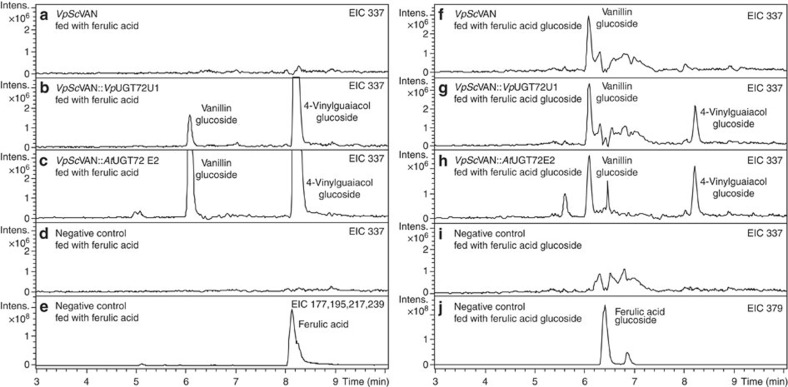
The ability of *VpSc*VAN expressed in an adapted yeast strain to metabolize ferulic acid and ferulic acid glucoside into vanillin and vanillin glucoside, respectively. (**a**) Expression of *VpSc*VAN does not result in formation of vanillin glucoside. Extracted ion chromatogram (EIC) 337: *m*/*z* vanillin glucoside (M+Na^+^). (**b**) Co-expression of *VpSc*VAN and *Vp*UGT72U1 results in formation of low amounts of vanillin glucoside. EIC 337: *m*/*z* vanillin glucoside (M+Na^+^). (**c**) Co-expression of *VpSc*VAN and *At*UGT72E2 results in formation of larger amounts of vanillin glucoside. EIC 337: *m*/*z* vanillin glucoside (M+Na^+^). (**d**) Negative control demonstrating that administration of ferulic acid to the adapted yeast strain does not result in vanillin glucoside formation. EIC 337: *m*/*z* vanillin glucoside (M+Na^+^). (**e**) Negative control monitoring the level of ferulic acid substrate administered. EIC 177, 195, 217, 239: *m*/*z* ferulic acid (M−OH=177, M+H^+^=195, M+Na^+^=217 and M+2Na^+^=239). Note that the EIC trace for vanillin glucoside (M+Na^+^=337) also monitors the presence of 4-vinylguaiacol glucoside (M+Na^+^=335) because this compound is present in such large amounts that the M+2 mass representing the natural isotope distribution is also recorded. (**f**) Expression of *VpSc*VAN results in formation of vanillin glucoside. EIC 337: *m*/*z* vanillin glucoside (M+Na^+^). (**g**) Co-expression of *VpSc*VAN and *Vp*UGT72U1 does not augment vanillin glucoside formation. EIC 337: *m*/*z* vanillin glucoside (M+Na^+^). (**h**) Co-expression of *VpSc*VAN and *At*UGT72E2 does not augment vanillin glucoside formation. EIC 337: *m*/*z* vanillin glucoside (M+Na^+^). (**i**) Empty vector control demonstrating that administration of ferulic acid glucoside to the adapted yeast strain does not result in vanillin glucoside formation. EIC 337: *m*/*z* vanillin glucoside (M+Na^+^). (**j**) Empty vector control monitoring the level of ferulic acid glucoside substrate administered. EIC 379: *m*/*z* ferulic acid glucoside (M+Na^+^). Note that the EIC trace for vanillin glucoside (M+Na^+^=337) also monitors the presence of 4-vinylguaiacol glucoside (M+Na^+^=335) because this compound is present in such large amounts that the M+2 mass representing the natural isotope distribution is also recorded. Intens., intensity.

In parallel with the experiment to test the activity of *Vp*VAN in *in vitro* transcription/translation assays, the catalytic activity of *Vp*VAN was further investigated by heterologous expression in *S. cerevisiae* (Fig. 5) using a range of putative substrates: *p*-coumaric acid, caffeic acid, ferulic acid, *p*-coumaric acid glucoside, caffeic acid glucoside, ferulic acid glucoside and feruloyl-CoA, *p*-coumaroyl-CoA and caffeoyl-CoA. For the yeast experiment, we have used the yeast codon-optimized *VpVAN* (*VpScVAN* gene sequence; Supplementary Fig. 2b). The specificity of the *VpSc*VAN enzyme was tested in yeast cells (Fig. 5) expressing vanillin synthase together with *Arabidopsis thaliana* UGT72E2 (*AtUGT72E2*) or together with *V. planifolia* UGT72U1 *(VpUGT72U1)*. *Vp*UGT72U1 is a novel UGT selected based on the combined *V. planifolia* transcriptomic and proteomic study (Supplementary Tables 1 and 2; Supplementary Data 1 and 2). *Vp*UGT72U1 specifically glucosylates vanillin (Supplementary Fig. 3), whereas *At*UGT72E2 has an extensive substrate specificity and is able to catalyse glucosylation of vanillin as well as the phenolic hydroxyl group of ferulic acid, *p*-coumaric acid, caffeic acid, *p*-hydroxybenzaldehyde and protocatechuicaldehyde.

Yeast has previously been reported to efficiently reduce vanillin to vanillyl alcohol^6^. The current studies were carried out using *S. cerevisiae* strain Y06460 in which alcohol dehydrogenase 6 (*ADH6*) is disrupted, because use of this strain circumvents reduction of the vanillin formed into vanillyl alcohol^6^. Genes were also integrated to simultaneously disrupt *EXG1* encoding an endogenous yeast exoglucosidase 1 (EXG1), which efficiently hydrolyses vanillin glucoside^6^. *VpVAN* was then stably integrated into the yeast chromosome either alone or together with *AtUGT72E2* or together with *VpUGT72U1*.

When *VpScVAN* was expressed alone in the yeast strain modified as described above in the presence of ferulic acid, no vanillin glucoside peak was observed in spite of the fact that the yeast endogenous exoglucosidase 1 (EXG1) had been knocked out. This demonstrated that the yeast does not contain a UGT able to glucosylate vanillin. Combined expression of *VpScVAN* and *VpUGT72U1* resulted in formation of vanillin glucoside when yeast was supplied with ferulic acid or ferulic acid glucoside. In addition, combined expression of *VpScVAN* and *AtUGT72E2* resulted in formation of vanillin glucoside when administrated with ferulic acid or ferulic acid glucoside. These studies demonstrate that the vanillin synthase is able to catalyse carbon double-bond cleavage of both ferulic acid and ferulic acid glucoside (Fig. 5).

As previously mentioned, *VpVAN* was predicted to encode a protein with an ER-targeting signal peptide. Accordingly, an additional series of biosynthetic studies were carried out with yeast harbouring stably integrated *AtUGT72E2* together with either *VpVAN* or *VpScVAN* or truncated *VpVAN* with no ER-targeting signal peptide (*vp*Δ*spvan*) or truncated *VpVAN* with no signal peptide and yeast codon optimized (*vpsc*Δ*spvan*). The yeast strains were incubated with putative substrates for 72 h and metabolite profiles determined by LC–MS. Formation of vanillin glucoside was observed with ferulic acid as substrate with *Vp*ΔSpVAN and *VpSc*ΔSpVAN (Supplementary Fig. 4). Thus *Vp*VAN is catalytically active towards ferulic acid in the presence as well as absence of the ER-targeting signal peptide. Carbon chain shortening of caffeic acid and *p*-coumaric acid or glucosides of these was not observed with any of the modified versions of *Vp*VAN.

Concomitant with the conversion of ferulic acid and its glucoside into vanillin and vanillin glucoside, yeasts are able to metabolize ferulic acid into 4-vinylguaiacol. The latter conversions are highly significant (Fig. 5). Two enzymes in *S. cerevisiae* are known to be responsible for the conversion of ferulic acid to 4-vinylguaiacol. These are phenylacrylate decarboxylase (PAD1) and ferulate decarboxylase (FDC1)^27^,^28^,^29^. Increased levels of *Vp*VAN-based vanillin production in yeast would thus be envisioned following disruption or downregulation of the two genes encoding PAD1 and FDC1.

**Figure 6:**
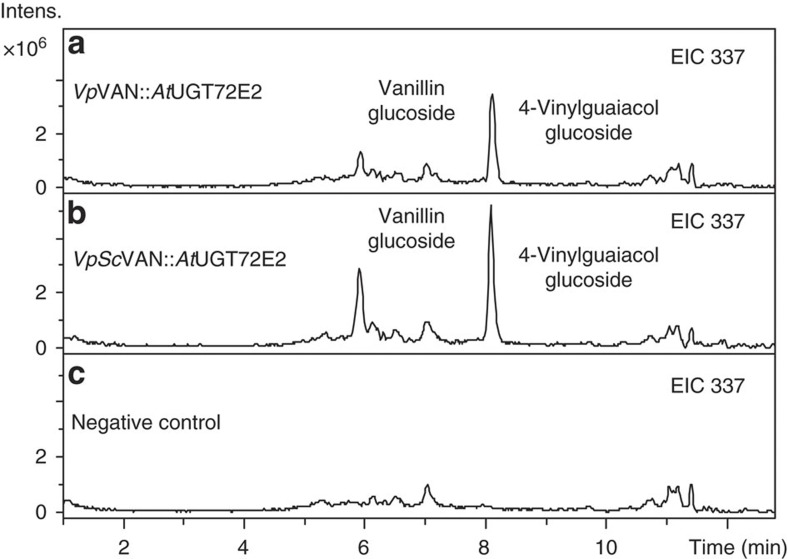
Vanillin glucoside formation in a molasses-based growth medium following incubation with yeast expressing *Vp*VAN or yeast codon-optimized *VpSc*VAN as well as *At*UGT72E2 stably integrated into the yeast genome. Both wild-type (panel **a**, EIC 337: *m*/*z* vanillin glucoside (M+Na^+^) and yeast codon-optimized versions (panel **b**, EIC 337: *m*/*z* vanillin glucoside (M+Na^+^) of *Vp*VAN were stably integrated into the yeast chromosome together with *AtUGT72E2*. Panel **c** is the wild type yeast strain (EIC 337: m/z vanillin glucoside (M+Na^+^)). The yeast strains were grown in Delft medium supplemented with 8% molasses and the production of vanillin glucoside was monitored by LC–MS and extracted ion chromatography. Intens., intensity.

Molasses are obtained as by-products in the production of sugar from sugar beets, sugar cane or sorghum, and these molasses contain ferulic acid^30^,^31^. To examine whether such material could be used for vanillin glucoside production, yeasts expressing *VpVAN* and *VpScVAN* as well as *AtUGT72E2* were grown in molasses-based growth medium using sugar beet as the source for the molasses. Vanillin glucoside formation was observed with both versions of *Vp*VAN, highlighting the potential of this enzyme for vanillin glucoside production based on inexpensive waste materials (Fig. 6).

### Establishing vanillin synthesis in tobacco and barley

**Figure 7:**
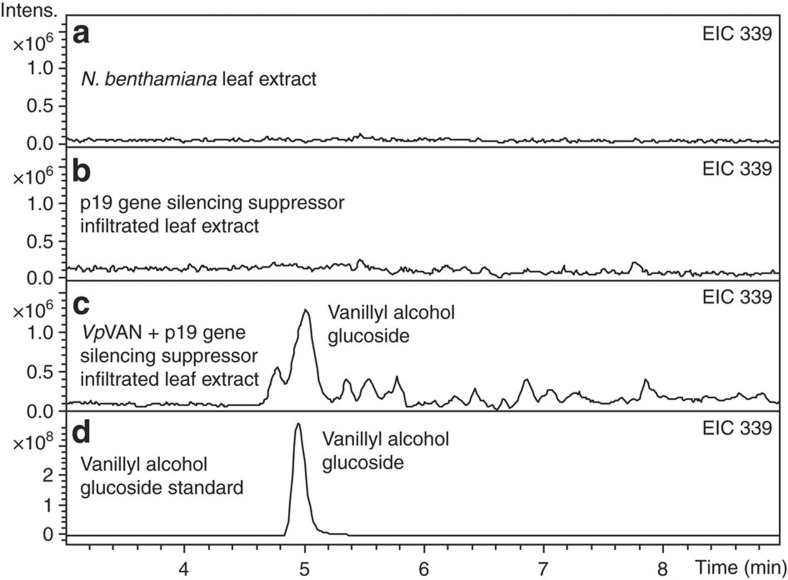
The biological activity of *Vp*VAN assessed by a transient expression in *Nicotiana benthamiana* as analysed by LC–MS and illustrated by extracted ion chromatogram (EIC). (**a**,**b**) Vanillyl alcohol glucoside is neither present in non-transformed *N. benthamiana* leaf extract nor in *p19* gene-silencing suppressor infiltrated leaf extract (EIC 339: *m*/*z* vanillyl alcohol glucoside (M+Na^+^) (**c**) *Vp*VAN was transferred to *Agrobacterium tumefaciens* and co-infiltrated with an *A. tumefaciens* strain harbouring the *p19* gene-silencing suppressor in *N. benthamiana* leaves. Four days after inoculation, the infiltrated tobacco leaves were harvested and subjected to metabolite profiling. EIC 339: *m*/*z* vanillyl alcohol glucoside (M+Na^+^). (**d**) Vanillyl alcohol glucoside standard. EIC 339: *m*/*z* vanillyl alcohol glucoside (M+Na^+^). Intens., intensity.

The catalytic activity of vanillin synthase in the presence and absence of a putative pro-peptide *in vivo* was analysed following transient expression in tobacco and stable expression in barley. The *in vivo* biological activity of *Vp*VAN (including the ER-targeting signal peptide) was assessed by transient expression in leaves of *N. benthamiana* in the absence of any exogenously added substrates. Gene constructs were transferred to *Agrobacterium tumefaciens* and co-infiltrated with an *A. tumefaciens* strain harbouring the *p19* gene-silencing suppressor. LC–MS profiling showed *Vp*VAN-dependent formation of vanillyl alcohol glucoside (Fig. 7). The vanillyl alcohol glucoside arises by reduction of vanillin by an alcohol dehydrogenase (E.C.1.1.1.1) and subsequent glucosylation of the primary alcohol group of vanillyl alcohol, as was previously observed in cell cultures of *N. plumbaginifolia*^32^ and yeast^6^. Biotechnological production of vanillin glucoside in plants other than *Vanilla* sp. by introduction of *Vp*VAN thus requires co-expression of a UGT that effectively glucosylates the free vanillin formed into the corresponding glucoside before its reduction into vanillyl alcohol.

**Figure 8:**
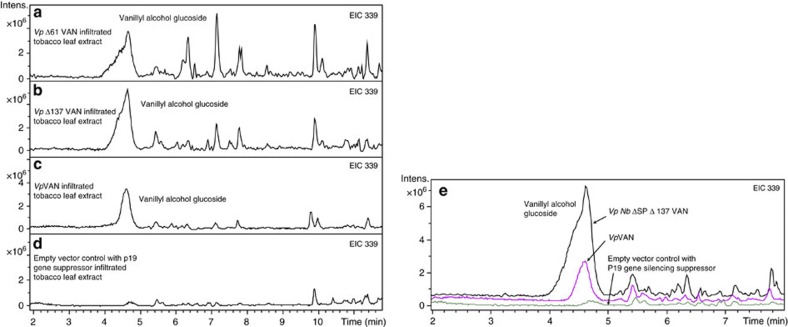
The biological activity of *Vp*VAN and modifications thereof assessed by a transient expression in *Nicotiana benthamiana*. (**a**) Vanillyl alcohol glucoside is present in the extract of leaves of *N. benthamiana* expressing *vp*Δ61*van.* EIC 339: *m*/*z* vanillyl alcohol glucoside (M+Na^+^); (**b**) Vanillyl alcohol glucoside is present in the extract of leaves of *N. benthamiana* expressing *vp*Δ137*van.* EIC 339: *m*/*z* vanillyl alcohol glucoside (M+Na^+^). (**c**) Vanillyl alcohol glucoside is present in the extract of leaves of *N. benthamiana* expressing *VpVAN.* EIC 339: *m*/*z* vanillyl alcohol glucoside (M+Na^+^). (**d**) Control using an empty vector construct harbouring the *p19* gene-silencing suppressor, EIC 339: *m*/*z* vanillyl alcohol glucoside (M+Na^+^). Modifications of the *Vp*VAN sequence have resulted in similar production of vanillyl alcohol glucoside in comparison with the amounts obtained from the *Vp*VAN sequence. (**e**) *vpnb*Δ*sp*Δ137*van* was transferred to *A. tumefaciens* and transiently expressed in tobacco. Four days after inoculation, the infiltrated tobacco leaves were harvested and subjected to metabolite profiling. EIC 339: *m*/*z* vanillyl alcohol glucoside (M+Na^+^). Modification of the *Vp*VAN sequence by insertion of the tobacco target sequence and pro-peptide protease cleavage site resulted in enhanced production of vanillyl alcohol glucoside in comparison with the amounts obtained from the *Vp*VAN sequence. Intens., intensity.

Transient expression of *vp*Δ137*van* and *vp*Δ61*van* was also included in this study to investigate the importance of secondary processing of *Vp*VAN for its *in vivo* activity. Introduction of each of these constructs encoding different truncated forms of *Vp*VA*N* was found to result in vanillyl alcohol glucoside production in similar amounts as observed with *Vp*VAN (Fig. 8a).

As previously mentioned, the *Vp*VAN sequence showed high sequence identity to proteins belonging to the family cysteine proteinases. We identified a protein belonging to the family of cysteine proteinases in tobacco in which the amino-acid sequence identity to *Vp*VAN was 71% (*N. benthamiana* cysteine proteinase gene sequence; supplementary Fig. 2c). In order to investigate the possibility to produce a nascent protein more amenable to proper targeting and processing by the endogenous tobacco machinery, a gene construct was made where the *Vp*VAN ER-targeting signal peptide and pro-peptide protease cleavage site were replaced with the putative signal peptide and the putative pro-peptide protease cleavage site from the tobacco cysteine protease (*vpnb*Δ*sp*Δ137*van* gene sequence; Supplementary Fig. 2d). The resulting construct *vpnb*Δ*sp*Δ137*van* was transferred to *A. tumefaciens* and transiently expressed in tobacco following infiltration. LC–MS profiling and extracted ion chromatography showed that modification of the *VpVAN* sequence by insertion of the tobacco target sequence and pro-peptide protease cleavage site resulted in a several fold higher production of vanillyl alcohol glucoside in comparison with the amounts obtained from the *VpVAN* sequence (Fig. 8e).

Plants belonging to the *Poaceae* family are known to accumulate higher amounts of ferulic acid and ferulic acid glucoside compared with other plant families^33^. It was therefore of interest to investigate the effects of stable *in vivo* expression of *VpVAN* in barley. In one series of transformations, the *VpVAN* gene sequence including the part encoding the ER-targeting signal peptide was codon optimized for barley (*VpHvVAN* gene sequence; Supplementary Fig. 2e). In a second series of transformations, the *VpHvVAN* gene sequence was additionally modified to encode a D-hordein signal peptide as a replacement for the original vanilla ER-targeting signal peptide (*vphv*Δ*spva*n). A constitutive ubiquitin promoter was used to drive the expression of both genes. Leaf samples from successfully transformed plants were collected 6–8 weeks after transfer of plantlets to the greenhouse and metabolic profiling was carried out by LC–MS. Barley plants transformed with *vphvΔspvan* were found to accumulate vanillyl alcohol glucoside in significantly higher levels than control plants (Supplementary Fig. 5).

### *Glechoma hederacea* contains a vanillin synthase homologue

**Figure 9:**
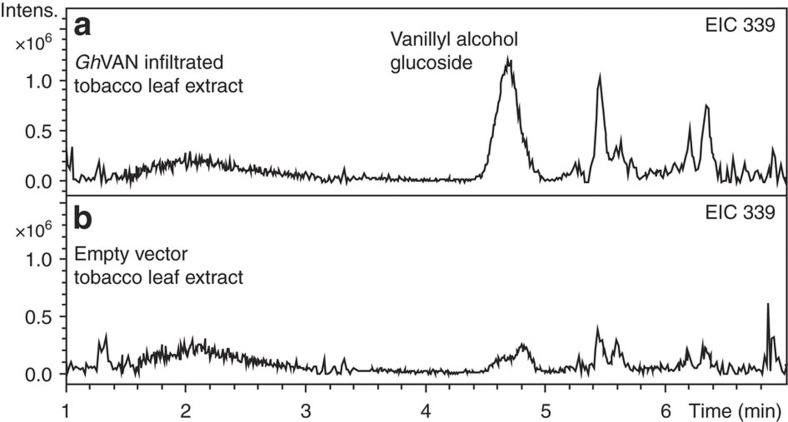
The biological activity of *Gh*VAN assessed by a transient expression in *Nicotiana benthamiana*. (**a**) Vanillyl alcohol glucoside is present in the extract of leaves of *N. benthamiana* expressing *GhVAN.* EIC 339: *m*/*z* vanillyl alcohol glucoside (M+Na^+^). (**b**) Control using an empty vector construct harbouring the *p19* gene-silencing suppressor. (EIC 339: *m*/*z* vanillyl alcohol glucoside (M+Na^+^). Intens., intensity.

A study of volatile constituents released from *G. hederacea* (ground ivy) belonging to the Lamiaceae family had shown that leaves of this plant release traces of vanillin^2^. RNA was isolated from the leaves. Transcriptome analysis identified an RNA sequence encoding a protein sequence showing 71% amino-acid sequence identity to *Vp*VAN. To investigate whether the ability to produce vanillin could be assigned to the expression of this gene, the gene was transiently expressed in tobacco. Analysis of the tobacco leaf extracts demonstrated that expression of the gene resulted in accumulation of vanillyl alcohol glucoside. Thus the gene encodes a protein with similar functional properties as *Vp*VAN. Accordingly the gene was assigned as *GhVAN* (Fig. 9) (*Gh*VAN sequence: Supplementary Fig. 2f).

## Discussion

**Figure 10:**
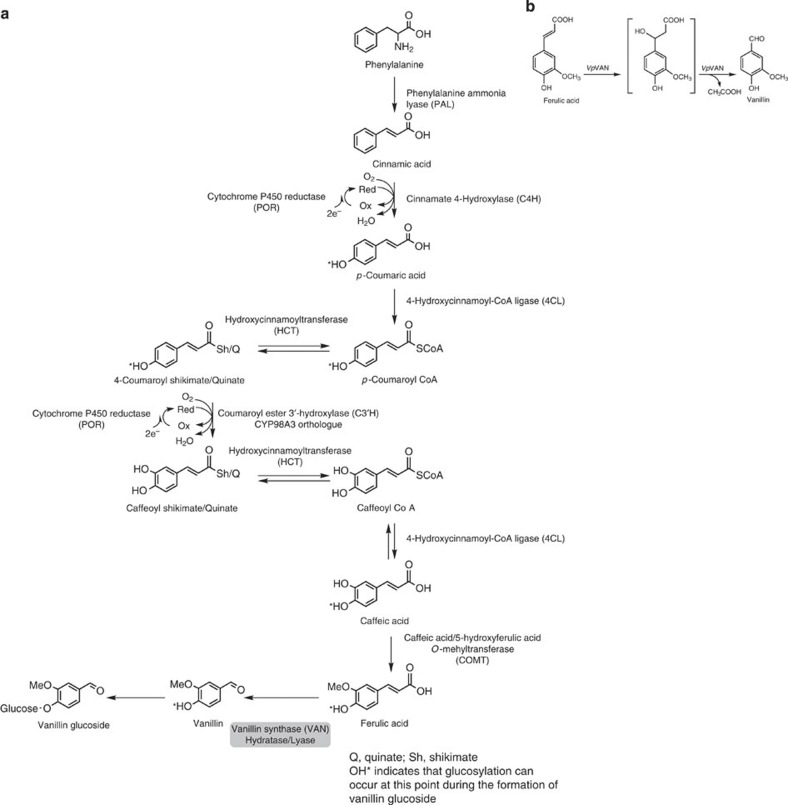
The *de novo* biosynthesis of vanillin is mediated by a single enzyme *Vp*VAN in the pods of *V. planifolia*. (**a**) The *de novo* biosynthesis of vanillin is mediated by a single enzyme, namely *V. planifolia* vanillin synthase (*Vp*VAN), which catalyses the two-carbon cleavage of ferulic acid and its glucoside to produce vanillin and vanillin glucoside, respectively. (**b**) The conversion of ferulic acid to vanillin is catalysed by *Vp*VAN and is envisioned to proceed sequentially by two partial reactions composed of an initial hydration addition reaction followed by a retro-aldol elimination reaction. Intens., intensity.

Numerous studies of the formation of vanillin and its glucoside have been carried out in the vanilla orchid *V. planifolia*, but no consensus biosynthetic pathway has emerged and specific enzymes involved in vanillin glucoside biosynthesis have not been conclusively demonstrated. In the current study, we have shown that the *de novo* biosynthesis of vanillin in the orchid *V. planifolia* and in *G. hederacea* (ground ivy), which belongs to the Lamiaceae family, is catalysed by a single enzyme, vanillin synthase that catalyses the two-carbon cleavage of ferulic acid and its glucoside to produce vanillin and vanillin glucoside, respectively (Fig. 10a). This conclusion was supported by biosynthetic experiments, which demonstrated that administration of the radiolabelled ferulic acid precursors phenylalanine and cinnamic acid to tissue slices of developing *V. planifolia* pods resulted in the formation of radiolabelled vanillin glucoside. These data combined with proteomic analysis demonstrated that vanillin biosynthesis takes place only in the inner part of the pod. This result is in accordance with previous observations^24^. Our *in situ* studies indicate that the vanillin synthase transcript and protein co-occur in the inner part of the pod corroborating the *in vivo* localization of the vanillin glucoside biosynthetic pathway.

Owing to the presence of high concentrations of 4-hydroxybenzaldehyde glucoside in mature pods and its structural similarity to vanillin glucoside, 4-hydroxybenzaldehyde or its glucoside has been proposed to be a precursor in the biosynthesis of vanillin and vanillin glucoside^19^. The huge pool of 4-hydroxybenzaldehyde glucoside found in mature fresh pods could either represent accumulation of an excess of 4-hydroxybenzaldehyde glucoside that is not yet converted into vanillin or reflect a separate function of 4-hydroxybenzaldehyde *per se*. In the current study, we have shown that administration of [^14^C] 4-hydroxybenzaldehyde to the inner part of the pod does not result in radiolabelling of vanillin glucoside under conditions where vanillin glucoside is known to be formed. The *in planta* biosynthetic routes to 4-hydroxybenzaldehyde and benzoic acid in other plant species also remain partly unresolved^17^,^34^. In the developing vanilla pod, the phenylalanine-derived phenylpropanoids such as *p*-coumaric acid, ferulic acid and sinapic acid may be directed towards formation of lignin monomers. The vanillin glucoside concentration increases with the age of the pod after pollination. The vanilla pod achieves its full-grown pod size about 3 months after pollination and is mature when about 10 months old. If left on the plant, the pod begins to split from the end, exposing its seeds. In the mature state, the pod only has a few requirements for *de novo* synthesis of cell wall components and the plant may therefore shift the flux of phenylpropanoid precursors from lignin biosynthesis to synthesis of vanillin glucoside and 4-hydroxybenzaldehyde glucoside in order to improve its potential for chemical defense of the maturing pod towards herbivores and pests. Vanillin and 4-hydroxybenzaldehyde exhibit anti-microbial properties. The metabolic changes in the course of pod ontogeny may thus serve to balance optimal pod development and defense.

The gene sequence that we have identified as encoding a vanillin synthase had previously been proposed to encode an enzyme, *p*-hydroxybenzaldehyde synthase (4-HBS), catalysing the conversion of *p*-coumaric acid into *p*-hydroxybenzaldehyde^19^. We tested the catalytic properties of the enzyme encoded by the gene sequence in coupled *in vitro* transcription/translation assays, following stable expression in yeast and following transient expression in tobacco and stable expression in barley. In the *in vitro* coupled transcription/translation system a range of putative substrates was provided including *p*-coumaric acid. In the transient and stable expression systems used, product formation was dependent on the availability of an endogenously produced substrate. In none of these experimental systems did we observe an ability of the *Vp*VAN enzyme to catalyse the conversion of *p*-coumaric acid into *p*-hydroxybenzaldehyde or *p*-hydroxybenzalcohol glucoside (*in planta*). This was monitored by LC–MS analyses and extracted ion monitoring. In all cases, the presence of a free or glycosylated hydroxyl group at the 4th position of the phenolic ring in combination with the presence of a methoxy group at the 3rd position was required for *Vp*VAN to exert activity. Experiments to measure the enzyme activity of the *Vp*VAN enzyme in protein extracts from the *V. planifolia* pod were not successful because the high amounts of endogenously produced vanillin glucoside and *p*-hydroxybenzaldehyde present prevented detection of minute additional amounts of product possibly formed following supplementation of precursors. Neither column chromatography nor dialysis for several days was sufficient to lower the amount of endogenous vanillin glucoside present to an acceptable level. In the study of Podstolski *et al.*^19^, different interconvertible isoforms of 4-HBS were partly purified from embryo cell cultures of *V. planifolia*. One of those isoforms, impurities present or residual amounts of endogenously bound *p*-hydroxybenzaldehyde or a simultaneously reported spontaneous background reaction may have given rise to the observed *p*-hydroxybenzaldehyde formation.

The conversion of ferulic acid and its glucoside into vanillin and the corresponding glucoside is envisioned to proceed sequentially by two partial reactions composed of an initial hydration addition reaction followed by a retro-aldol elimination reaction (Fig. 10b). The initial reaction consists in the addition of water to the double bond. The β-hydroxy carboxylic acid formed then undergoes a well-known retro-aldol elimination reaction, which results in the formation of vanillin and acetic acid in stoichiometric amounts. Since our studies excluded the requirement for any cofactors, this remains the only plausible reaction mechanism, although we did not carry out assays to determine the acetate release.

This reaction mechanism has been demonstrated in some bacteria for the bioconversion of the CoA thioester of ferulic acid to vanillin, for example, in cultures of *Pseudomonas fluorescens* by the enzyme 4-hydroxycinnamoyl-CoA hydratase/lyase (HCHL). HCHL catalyses the degradation of a range of 4-hydroxycinnamic acid CoA thioesters including ferulic acid-CoA^35^,^36^. This bacterial enzyme is a member of the low sequence similarity hydratase/isomerase superfamily of enzymes also referred to as the crotonase superfamily. Enzymes belonging to this family are known to catalyse highly divergent types of reactions including hydratase/lyase reactions, and the specific function of individual family members cannot easily be deduced solely from their amino-acid sequence because the amino-acid residues specifying the activity are scattered throughout the entire protein sequence^37^. The intermediates in these reactions are usually thioester enolate anions stabilized by a conserved oxyanion hole through hydrogen bonds^38^. Two Glu residues serve as acid/base catalysts for the reaction, although in some members of the crotonase family the second Glu residue is absent^38^. A sequence alignment of *Vp*VAN with the HCHL sequence from *P. fluorescens* shows insignificant sequence similarity. Of the 55 conserved amino-acid residues scattered over the entire protein sequence in selected bacterial sequences belonging to the crotonase superfamily (see Achterholt *et al.*^39^, Fig. 5), only 11 were similarly positioned in *Vp*VAN. The alignment with *Vp*VAN identifies the position of the two consensus sequences involved in stabilizing the oxyanion hole in the crotonase superfamily. These show low sequence identity YGSEEE (residues 67–72) and QGI (residues 147–149) to the consensus sequences found in crotonases^38^.

A general sequence identity search using GenBank showed that the *Vp*VAN protein sequence has a much higher sequence identity to cysteine proteinases. Cysteine proteinases are expressed as a pre-protein with an N-terminal ER-targeting signal peptide being part of a pro-peptide domain containing 130–160 residues^40^. In the mature protein, the pro-peptide sequence is removed either with the aid of a processing enzyme or auto-catalytically^41^. Autocatalytic cleavage would have resulted in the formation of a protein with a mass of 23.89 kD. The *in vitro* transcription/translation experiments in which the *Vp*VAN protein formed was labelled with ^35^S methionine showed no evidence of autocatalytic processing (Fig. 2b) indicating that removal of the pro-peptide requires the action of a separate processing enzyme. Residues that are known to be conserved among different cysteine proteinases because they form part of the active site were also found to be conserved in *Vp*VAN. These include Q156, C162, N301 and NSW322-24 (ref. 42). Likewise the six cysteine residues known to be involved in disulphide bridge formation in cysteine proteinases are conserved in *Vp*VAN: C159/C202 C193/C235, C293/C343 (ref. 42). The non-contiguous ERFNIN signature (E × 3R × 3F × 3N × 3I/V × 3N) found in the pro-peptide of some groups of papain-like cysteine proteinases is also present in *Vp*VAN (E72, R76, F80, N83, I87 and N91). The GC × GG domain known from papaine-like cysteine proteinases is also conserved in *Vp*VAN (residues 201–205; Supplementary Fig. 2g). A putative sumoylation site FKME is located near the C-terminal end of *Vp*VAN (residues 334–337 Supplementary Fig. 2g). SUMO modification of a tomato cysteine proteinase targeted the cysteinase to the nucleus where it activated a gene in the ethylene biosynthetic pathway^43^. This may imply a role of *Vp*VAN in vanilla pod senescence. The pro-peptide released may act as an inhibitor of plant pests^44^,^45^. The catalytic mechanism of cysteine proteinases involves formation of a tetrahedral transition state composed of an oxyanion hole stabilized by hydrogen bonds. In the processed mature cysteine proteinase papain, the backbone amide of the catalytic C25 residue and the side chain amide of Q19 provide the hydrogen bonds^46^. These residues correspond to residues C162 and Q156 in *Vp*VAN. The establishment of a transition state composed of an oxyanion hole stabilized by hydrogen bonds is thus a common feature of those two enzyme classes to which *Vp*VAN shows sequence homology. We therefore propose that formation of a similar oxyanion transition state constitutes the initial step in the *Vp*VAN catalysed conversion of ferulic acid and its glucoside into vanillin and vanillin glucoside, respectively. The oxyanion intermediate would facilitate hydration and constitute the intermediate that by a retro-aldol elimination reaction affords C–C cleavage of the propanoid side chain as required for vanillin formation.

Two putative protease cleavage sites in *Vp*VAN were identified after residue 61(RFAR/RYGK) and residue 137 (VDGV/LPVT). The N-terminal pro-peptide sequence in *Vp*VAN as well as in plant cysteine proteinases may be envisioned to serve different functions. The pro-peptide sequence may control proper intracellular targeting, may promote proper folding of the mature enzyme and may also serve to maintain the enzyme in an inactive form in the cell to balance its function according to physiological demands. In our studies, we have observed that *Vp*VAN is also active in the presence of the pro-peptide sequence (Figs 3, 5a,b and 6 and so on) documenting that the presence of the pro-peptide does not severely inhibit *Vp*VAN activity. However, when the *Vp*VAN pro-peptide was replaced with the ER-targeting putative signal peptide and the putative pro-peptide protease cleavage site from the tobacco cysteine protease and transiently expressed in tobacco, higher levels of vanillin alcohol glucoside were obtained compared with parallel experiments with *Vp*VAN. It is possible that the presence of the tobacco ER-targeting signal peptide and the pro-peptide protease cleavage site from the tobacco is able to target *Vp*VAN to the correct cell compartment ensuring correct folding and post-translation modification of the protein, resulting in enhanced enzyme activity.

The identification of a hydratase/lyase type enzyme as being a vanillin synthase offers new opportunities for the *Vanilla* pod-based industries. The accumulation of vanillin glucoside in the capsules of cultivated vines in response to environmental challenges may now be assessed at the molecular level. Likewise, the basis for development of genetic markers for the selection of vanilla orchid varieties with improved aromatic properties has now been laid down. Vanillin produced biologically is termed ‘natural’ vanillin and has a high economic value compared with chemically synthesized vanillin. Likewise, in the transition towards a bio-based economy, it is important to develop sustainable production systems to replace those currently based on fossil fuels. The demonstration that a single enzyme in the vanilla pod catalyses the conversion of ferulic acid and ferulic acid glucoside into vanillin and vanillin glucoside provides several options for biotechnological applications. As demonstrated in the current study, molasses may be used for vanillin production based on their ferulic acid content and following supplementation with yeast expressing vanillin synthase, but are devoid of ferulate decarboxylase activity. Ferulic acid is a key intermediate in lignin monomer formation in plants, so stable expression of *VpVAN* and, for example, *AtUGT72E2* in plants would be expected to result in vanillin glucoside formation in varying amounts. In cured *Vanilla* pods, only partial hydrolysis of the vanillin glucoside originally present has occurred offering a slow release aroma effect when residual amounts of the glucoside are hydrolysed by microbial enzymes in the mouth saliva following human ingestion. In pig production farms, addition of vanillin to the pig feed has been shown to increase fodder uptake by the pigs and their growth rate^47^. If so desired, transgenic plants with high vanillin synthase activity may be used as production sources for vanillin glucoside. Alternatively, yeast and other microorganisms may be used as production platforms following stable integration of genes encoding for enzyme conversion of phenylalanine into ferulic acid, vanillin synthase and a vanillin glucosyltransferase.

## Methods

### Plant material

Healthy branches of *V. planifolia* carrying foliage and green vanilla pods were harvested at La Réunion 3 and 6 months after pollination and were shipped by courier carrier to Denmark while maintaining high-humidity conditions. Pod discs stabilized in RNAlater (Qiagen) were also imported from the biological resource center, VATEL, CIRAD, Saint-Pierre, La Réunion, France.

*N. benthamiana* plants (3 weeks old) were used for the transient expression of *Vp*VAN.

The spring barley cultivar Golden Promise was grown in growth cabinets at a day/night temperature regime of 15/10 °C with a 16-h light period (light intensity: 350 μE m^−2^ s^−1^). Immature embryos were isolated 12–14 days after pollination and used for Agrobacterium-mediated production of stably transformed barley plants.

### Strains and growth media

*Escherichia coli DH5α* (*endA1*, *hsdR17*, *gyrA96*, *thi-1*, *relA1*, *supE44*, *recA1*, *ΔlacU169* (*Φ80 lacZΔM15*)) was used as the recipient strain for cloning experiments and plasmid propagation. It was grown following standard procedures.

*S. cerevisiae* strain Y06460 (Euroscarf) (BY4741; Mat a; his3D1; leu2D0; met15D0; ura3D0; YMR318c::kanMX4) and derivatives were grown in yeast extract peptone dextrose media, Delft medium supplemented with sugar beet molasses and appropriate synthetic complete (SC) media.

Agrobacterium strain AGL1 was used for the transient and stable expression assays *in planta* and grown following standard procedures in LB medium with appropriate antibiotics.

### Transcriptomic analysis

Total RNA from *V. planifolia* was prepared from ~100 mg of 6-month-old vanilla pod tissue using the RNeasy plant mini kit (Qiagen, http://www.qiagen.com) with on-column DNase I digestion. Quality of RNA was assessed using a bioanalyzer (Agilent). RNA (about 100 μg total) was provided to Eurofins MWG Operon ( www.eurofinsdna.com) for 454 Roche sequencing. A normalized assembly of sequences was obtained. Obtained sequencing reads were *de novo* assembled using CLC Genomics Workbench 5.0 with default settings. Raw sequencing reads were submitted to the Sequence Read Archive (SRA) database at National Center for Biotechnology Information (Bioproject accession no. SRP023166). Total RNA from *Glechoma hederacea* was isolated using the RNeasy plant kit (Qiagen) and provided to Macrogen ( www.macrogen.com) for Illumina HiSeq sequencing.

Raw sequencing reads were submitted to the Sequence Read Archive database at the National Center for Biotechnology Information (accession no. KJ775791).

### Proteomic analysis

The inner part of the vanilla pod was ground to a fine powder in liquid nitrogen and extracted in 400 mM Tris/HCl (pH 8), 20 mM MgCl_2_. Solubilized proteins were separated by SDS–PAGE on 10% Tris-glycine gels (Bio-Rad) and stained using Coomassie Brilliant Blue R-250 (Bio-Rad). Protein masses were estimated using a standard broad range Bio-Rad molecular mass marker. In-gel digestion of protein bands and MS were performed by the University of Victoria—Genome BC Proteomic center (method information found on www.proteincentre.com^48^). The peptide mass data and tandem mass data obtained were used to search known protein sequences from the *V. planifolia* transcriptome data.

### Isolation and subcloning of genes

A complementary DNA (cDNA) library made from a 6-month-old *V. planifolia* pod was kindly provided by Evolva A/S Denmark. The cDNA library was inserted in a pYES2 vector (Invitrogen) ( http://tools.invitrogen.com/content/sfs/manuals/pyes2_man.pdf). cDNA from *Glechoma hederacea* was made from material sourced in Basel, Switzerland. Total RNA was isolated using the RNeasy plant kit (Qiagen) and cDNA was made using the Mint2 cDNA synthesis kit (Evrogen) ( www.evrogen.com).

Candidate genes identified from the transcriptome data were amplified from the cDNA library by PCR with gene-specific primers (Supplementary Tables 1 and 3) to obtain full-length sequences. The PCR products were subsequently cloned in blunt-II-topo vector (Invitrogen) in *E. coli*. Plasmids were purified using the miniprep kit (Qiagen) and gene sequences were confirmed by sequencing.

### Construction of expression cassettes

Yeast expression plasmids were constructed by transferring gene inserts by restriction digestion cloning with the suitable restriction enzymes and subsequently ligated into the yeast expression vector p426-GPD, containing the constitutive *GPD* promoter and the p416-TEF vector containing the constitutive *TEF* promoter^49^ (Supplementary Table 1). Restriction enzymes and T4 ligase were from New England BioLabs and reactions were carried out according to manufacturers’ instructions. Vectors for chromosomal integration of genes were constructed using the uracil-specific excision reagent (USER) cloning method and a vector system adapted from Mikkelsen *et al.*^50^ A plasmid (pVAN714) containing UP- and DOWN-targeting fragments for replacing the yeast endogenous exoglucosidase 1 gene (*EXG1*) was constructed similarly as described in Mikkelsen *et al.*^50^ The genes and a fused *TEF1/PGK1* promoter DNA fragment were USER cloned into pVAN714.

Plant expression vectors for tobacco transient expression were constructed using Gateway cloning technology (Invitrogen). The cDNAs of interest were PCR amplified with gene-specific primers with *att*B overhangs; 5′-GGGGACAAGTTTGTACAAAAAAGCAGGCTAAAAATGTCTATGGCAGCTAAGCTCCTCTTC-3′ and 5′-GGGGACCCAGCTTTCTTGTACAAAGTGGTCACAGCCACAATGGGATAAGATG-3′ (All primers are listed in Supplementary Table 3) and cloned in the pDONR207 gateway vector (Invitrogen) and subsequently transferred to the destination vector pJAM1502 (ref. 51) by homologous recombination.

Plant expression vectors for tobacco transient expression to test the catalytical activity of *Vp*VAN and different modifications thereof were constructed using the Gateway cloning technology (Invitrogen). The cDNAs of interest were PCR amplified with *att*B overhangs; *Vp*Δ61*van* 5′-GGGGACAAGTTTGTACAAAAAAGCAGGCTTCAAAAATGTCTTCGATGAGGTACGGGAAGAGCTACGGATCGGAG-3′ and 5′-GGGGACCACTTTGTACAAGAAAGCTGGGTCTACACAGCCACAATGGGATAAG-3′, *Vp*Δ137*van* 5′-GGGGACAAGTTTGTACAAAAAAGCAGGCTTCAAAAATGTCTTCGATGGCGTGCTTCCTGTAACGAGGGA-3′ and 5′-GGGGACCACTTTGTACAAGAAAGCTGGGTCTACACAGCCACAATGGGATAAG-3′, and cloned in the pDONR207 gateway vector (Invitrogen) and subsequently transferred to the destination vector pEAQ-HT-DEST3 (ref. 52) by homologous recombination. *vpnbΔspΔ137van* (sequence: Supplementary Fig. 2e) was synthetically synthesized by MWG-Biotech ( www.mwg-biotech.com). Empty vector control was the destination vector pEAQ-HT-DEST3 (ref. 52) having a nonsense gene with a few amino acids having a start and a stop codon.

The binary vector pUCEUBI:SP-USER:NOS was used for stable transformation of Barley. Engineering of the construct was done as described previously^53^. In short, *Vp*VAN was amplified by PCR using the specific primers: *VpHvVAN* 5′-GGTCTTAAUATGGCAGCTAAGCTCCTC-3′ and 5′-GGCATTAAUTCAAACAGCCACAATGGGGTATG-3′ and *VpHvΔspvan* 5′-GGTCTTAAUATGTTCCTGCTGTTTCTAGTGTCCG-3′ and 5′-GGCATTAAUTCAAACAGCCACAATGGGGTATG-3′. The PCR-reactions were carried out using PfuTurbo CX Hotstart DNA polymerase (Stratagene) according to the manufacturer’s instructions. The resulting PCR product was inserted into the binary vector by using USERTM cloning as described previously^53^.

### *In vitro* transcription/translation

The TNT Quick Coupled Transcription/Translation kit for PCR-generated DNA (Promega) was used to produce proteins of interest directly from PCR products. L-[^35^S]-Methionine was included to permit monitoring of the radiolabelled proteins formed following separation by SDS–PAGE and visualized by incubating dried gels for 48 h on phosphorimager screens, which were scanned with a STORM 860 molecular imager (Molecular Dynamics).

### *In vitro* protein assay

Proteins produced in coupled *in vitro* transcription/translation assays were analysed for their enzyme catalytic abilities by incubation of aliquots (10 μl) with 0.5–5 mM of the following substrates: ferulic acid (Sigma), *p*-coumaric acid (Sigma), caffeic acid (Sigma), ferulic acid glucoside, *p*-coumaric acid glucoside, caffeic acid glucoside, caffeoyl-Coenzyme A (MicroCombiChem e.K.), *p*-coumaryl-Coenzyme A (MicroCombiChem e.K.), feruloyl-Coenzyme A (MicroCombiChem e.K.) or sinapyl-Coenzyme A (MicroCombiChem e.K.) in 400 mM Tris/HCl (pH 8), 20 mM MgCl_2_ and 2.5 mM dithiothreitol (total volume: 50 μl). Enzyme assays were carried out in the presence and absence of 2.5 mM dithiothreitol, 0.1 mM ATP and 0.1 mM NAD^+^. Aliquots (10 μl) were withdrawn at specific time points and enzyme activity stopped by MeOH addition (25 μl, 25% (v/v)) and heating (45 °C, 15 min). Samples were cooled on ice (30 min), centrifuged (7,400*g*, 10 min) in microtitre filter plates (Merck Millipore) and the filtrate was finally analysed by LC–MS.

### Yeast transformation

Yeast expression plasmids with candidate genes were transformed into *S. cerevisiae* using the LiAc/SS carrier DNA/polyethylene glycol method^54^. All transformants were grown on SC medium lacking Uracil (URA) to select positive transformants (yeast strains constructed—Supplementary Table 2).

### Enzyme assays using yeast

Transformed yeast cells were cultured in 50 ml of liquid SC-URA for 24 h. Putative substrates (2.5 mM) were administered to the yeast culture (3 ml) and incubated (28 °C, 72 h, 200 r.p.m.) using a sterile 24-well microtitre plate (Biopioneer Inc). Incubation was stopped by addition of MeOH (350 μl, 35% (v/v)) and the samples prepared for LC–MS as described above.

### Transient expression of *Vp*VAN in tobacco

Overnight cultures of an *Agrobacterium tumefaciens* strain AGL1 containing either the recombined pJAM1502 or pEAQ-HT-DEST3 vectors harbouring the *VpVAN* cDNA and an *A. tumefaciens* strain AGL1 carrying the recombined pJAM1502 vector harbouring the gene-silencing inhibitor protein 19 (p19)^34^ were harvested by centrifugation and resuspended (OD_600_=2.0) in 10 mM MES pH 5.5, 10 mM MgCl_2_ and 100 μM acetosyringone. After incubation (4 h, room temperature), the two *A. tumefaciens* strains were used to co-infiltrate leaves of 3-week-old *N. benthamiana* plants grown at 24 °C (day) and 17 °C (night). After 4 or 5 days, leaf discs (1 cm diameter) were stamped out from the infiltrated leaves and metabolites extracted in 60% (v/v) MeOH for LC–MS analysis.

### Stable expression of *VpHv*VAN in barley

The vectors harbouring *VpHvVAN* and *VpHvΔspvan* cDNA (as described previously) were transformed into *A. tumefaciens* strain AGL0 using the freeze/thaw method and selected on medium with 50 mg l^−1^ spectinomycin and 25 mg l^−1^ rifampicin. Immature embryos isolated from barley plants 12–14 days after pollination were used for Agrobacterium-mediated transformation following the procedure described elsewhere^55^. Regenerated transgenic plants were transferred to the greenhouse. Leaf samples were collected 6–8 weeks after transfer to the greenhouse.

### Biosynthetic assays with green vanilla pod discs

Fresh vanilla pods were harvested 3 and 6 months after pollination. The pods were cut into small discs using a scalpel and further dissected to separate the inner and outer part of the pod. Radiolabelled precursors (0.5 μCi) were administered to samples representing the inner and outer part of the pod (approx. identical fresh weight) and embedded (30 °C) in 400 mM Tris/HCl pH 8, 20 mM MgCl_2_ for 24 h.

### In tube *in situ* PCR using tissues of vanilla pod discs

Fresh vanilla pods from *V. planifolia* were cut into small pieces (2–4 mm^3^) and immediately fixed (4 h, 4 °C) in freshly prepared aqueous FAA (2% (v/v) formaldehyde, 5% (v/v) acetic acid, 63% (v/v) ethanol in phosphate-buffered saline). The transcript level of *VpVAN* in different cell types was visualized based on specific primers^56^; *in situ Vp*VAN 5′-AAGCCTTTGAATACGTTAAGTACAATGGA-3′ and *in situ Vp*VAN reverse 5′-GTGTCACTGCTGTATACACCTTTCTT-3′.

### Analytical chemistry

The ^14^C-labelled products formed in biosynthetic experiments with fresh vanilla pods as well as in *in vitro* protein assays were applied to Silica Gel 60 F254 TLC plates (Merck, http://www.merck-chemicals.com). The plates were developed in ethyl acetate: acetone: dichloromethane: methanol: water (40:30:12:10:8, v/v/v/v/v), dried, exposed (48 h) on phosphorimager screens (Molecular Dynamics, http://www.moleculardynamics.com) and the radiolabelled compounds formed were visualized using a Storm 860 Molecular Imager (Molecular Dynamics). Identification of the radiolabelled compounds formed was guided by co-application of authentic standards. Unambiguous structural verification of the products formed was obtained using LC–MS including accurate mass determination and comparison of retention times and fragmentation patterns with those of authentic reference compounds^57^.

### Chemical synthesis

*p*-Hydroxybenzaldehyde glucoside and vanillyl alcohol glucoside: the corresponding aglycons were glucosylated using 2,3,4,6-tetra-*O*-acetyl-α-D-glucopyranosyl bromide. The reaction was performed in aqueous organic basic media using homogeneous reaction conditions and aqueous NaOH with acetone as the organic co-solvent following the method reported by Mauthner^58^. The target glucosides were obtained by Ze´mplen deactylation of the aryl *O*-protected glucosides.

Vanillic acid glucoside: vanillin *O*-protected glucoside was synthesized as mentioned for *p*-hydroxybenzaldehyde glucoside and oxidized to the corresponding carboxylic acid using potassium permanganate (KMnO_4_). Deacetylation was accomplished as reported above.

4-β-D-glucopyranosylcoumaric acid: *p-*coumaric acid glucoside and ferulic acid glucoside were chemically synthesized according to Galland *et al.*^59^ and references therein.

Ferulic acid glucoside^13^C/^14^C_6_ analogue: to synthesize the desired ferulic acid-^13^C_6_/^14^C_6_-β-D-glucoside, the key step is the glycosylation of methyl ferulate by 1-fluoro-2,3,4,6-tetra-*O*-acetyl-D-glucopyranose-^13^C_6_/^14^C_6_ with the BF_3_.Et_2_O complex as the activator^60^. In this work, D-glucose-13C6/14C6 in the ratio of 98:2 was converted into the corresponding glycosyl fluoride as reported previously^61^.

The purity and structural conformation of the synthesized compounds were verified by NMR spectroscopy. In all cases, the ^1^H- and ^13^C-NMR chemical shifts for the chemically synthesized target molecules were consistent with previously reported data: *p-*coumaric acid glucoside (4-β-D-glucopyranosylcoumaric acid) and ferulic acid glucoside (4-β-D-glucopyranosylferulic acid) as reported by Galland *et al.*^59^ and references therein, *p*-hydroxybenzaldehyde glucoside (4-β-D-glucopyranosylbenzaldehyde)^62^, vanillyl alcohol glucoside (4-β-D-glucopyranosylvanillyl alcohol)^63^ and vanillic acid glucoside (4-β-D-glucopyranosylvanillic acid)^64^.

## 

## Author contributions

N.J.G. performed the [^14^C]-radiolabelled precursor feeding experiments and TLC analysis, mRNA extractions for 454 transcriptome sequencing and handling 454 sequence data, proteomic studies and handling proteomic data, *in vitro* transcription/translation studies, all the molecular biology analysis, yeast strain constructions, tobacco expression studies, in tube *in situ* PCR and contributed to writing the manuscript. E.H.H. constructed all the yeast integration plasmids, contributed to planning, designing the project and writing the manuscript. R.K. contributed to performing the proteomic studies and writing the manuscript. C.E.O. carried out the LC–MS analysis. M.S.M. designed and performed the chemical synthesis of all glucoside substrates. K.J. sectioned the vanilla pod for in tube *in situ* PCR and performed the fluorescence microscopy. M.G. contributed to writing the manuscript and was in charge of courier shipment of fresh vanilla materials from La Réunion to Denmark. I.H. and K.H contributed to the planning and performance of the barley transformation. B.L.M. planned and designed the project, provided biochemical expertise and scientific mentoring and contributed to writing the manuscript.

## Additional information

**Accession codes:** Raw sequencing reads of *V. planifolia* transcriptome is submitted to the Sequence Read Archive (SRA) database at National Center for Biotechnology Information (Bioproject accession no. SRP023166). The raw sequencing data reads have been deposited in the Sequence Read Archive under accession code KJ775791.

**How to cite this article:** Gallage, N. J. *et al.* Vanillin formation from ferulic acid in *Vanilla planifolia* is catalysed by a single enzyme. *Nat. Commun.* 5:4037 doi: 10.1038/ncomms5037 (2014).

## Supplementary Material

Supplementary Figures and Tables Supplementary Figures 1-5 and Supplementary Tables 1-3

Supplementary Data 1 Protein hits information from MASCOT. Identification of proteins in vanillin biosynthesis pathway by searching LC-MS/MS data against DNA conreads of pyrosequencing data and protein sequences that belonging to intrested enzyme families from other plants.

Supplementary Data 2 Summary of database search for identification of proteins in the Vanilla Planifolia inner part crude protein extract by searching the pyrosequencing DNA databases of candidate enzyme families in all reading frames and protein data bases including candidate enzyme families from other plant families
