# Supplementary material for: Vanillin formation from ferulic acid in Vanilla planifolia is catalysed by a single enzyme
Source: Nat Commun. 2014 Jun 19;5:4037. doi: 10.1038/ncomms5037 (PMC4083428; doi:10.1038/ncomms5037)
Supplement: Supplementary Figures and Tables — Supplementary Figures 1-5 and Supplementary Tables 1-3 [file ncomms5037-s1.pdf]

Supplementary Figure 1: Unlabeled standards analysed with TLC

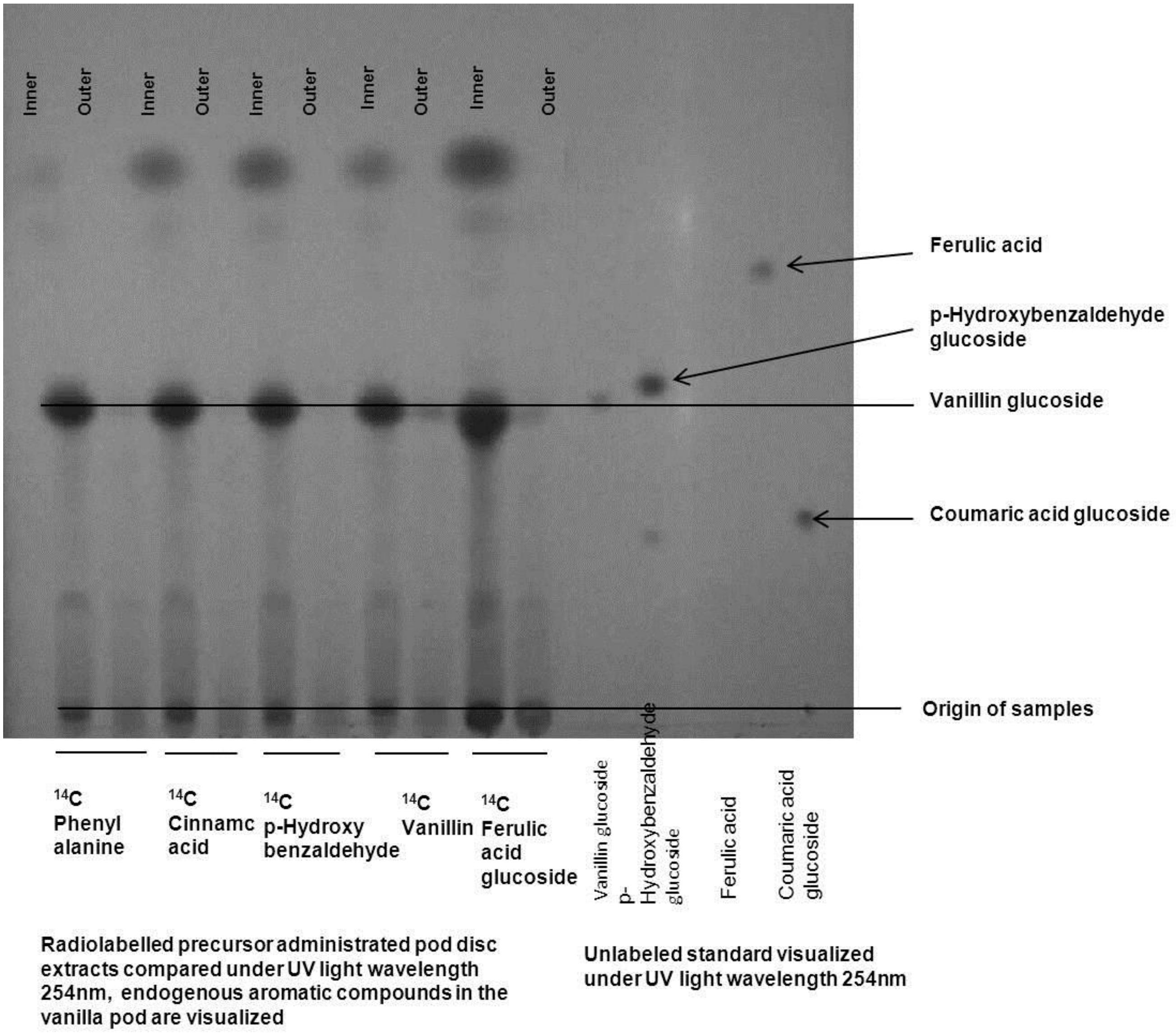

Standards for TLC analysis are visualized under UV light at wavelength 254nm

All fresh vanilla pod discs samples administrated with ^{14}C precursors were spotted on a TLC plate with non-^{14}C labeled standards**.** The ascending patterns were compared under UV light wavelength 254nm. Radioactively labeled compounds are too dilute to be visualized by UV, yet it is possible to visualize the metabolite profile of aromatic compounds in the inner and outer parts of the vanilla pod. Supplementary figure 1 shows that there is a large quantity of endogenous vanillin glucoside present in all vanilla pod samples and as expected, the inner part of the pod contains a significantly larger amount of vanillin glucoside than the outer part. The figure (to the right) shows that *p*- hydroxybenzaldehyde glucoside migrates above vanillin glucoside on the TLC with the solvent system used.

Supplementary Figure 2:

a) *VpVAN* gene sequence

ATG GCA GCT AAG CTC CTC TTC TTC CTA CTC TTC CTG GTC TCC GCC CTC TCC GTC GCG CTC GCC GGT TTC GAA GAA GAC AAT CCA ATC CGG TCC GTT ACA CAA AGG CCT GAC TCG ATT GAG CCT GCC ATC CTC GGC GTC CTT GGC AGT TGC CGC CAC GCC TTC CAC TTC GCA CGG TTC GCC CGC AGG TAC GGG AAG AGC TAC GGA TCG GAG GAG GAG ATC AAG AAG AGG TTC GGG ATC TTC GTG GAG AAT CTA GCG TTT ATC CGG TCC ACT AAT CGG AAG GAT CTG TCG TAT ACC CTA GGA ATC AAC CAA TTC GCC GAC CTG ACC TGG GAG GAA TTC CGG ACC AAT CGC CTT GGT GCG GCG CAG AAC TGC TCG GCG ACT GCG CAT GGA AAC CAC CGG TTT GTC GAT GGC GTG CTT CCT GTA ACG AGG GAT TGG AGG GAG CAA GGG ATA GTG AGC CCT GTA AAG GAC CAA GGA AGC TGT GGA TCT TGC TGG ACT TTC AGT ACT ACT GGA GCA CTA GAG GCT GCA TAT ACA CAG CTA ACT GGA AAG AGC ACA TCA TTA TCT GAA CAG CAA CTT GTG GAC TGT GCC TCA GCA TTC AAT AAC TTT GGA TGC AAT GGA GGT TTG CCT TCC CAA GCC TTT GAA TAC GTT AAG TAC AAT GGA GGC ATC GAC ACA GAA CAG ACT TAT CCA TAC CTT GGT GTC AAT GGT ATC TGC AAC TTC AAG CAG GAG AAT GTT GGT GTC AAG GTC ATT GAT TCG ATA AAC ATC ACC CTG GGT GCT GAG GAT GAG TTG AAG CAT GCA GTG GGC TTG GTG CGT CCA GTT AGC GTT GCA TTT GAG GTT GTG AAA GGT TTC AAT CTG TAC AAG AAA GGT GTA TAC AGC AGT GAC ACC TGT GGA AGA GAT CCA ATG GAT GTG AAC CAC GCA GTT CTT GCC GTC GGT TAT GGA GTC GAG GAC GGG ATT CCT TAT TGG CTC ATC AAG AAC TCA TGG GGT ACA AAT TGG GGT GAC AAT GGC TAC TTT AAG ATG GAA CTC GGC AAG AAC ATG TGT GGT GTT GCA ACT TGC GCA TCT TAT CCC ATT GTG GCT GTG TAG

b) *VpScVAN* codon optimized for yeast expression

ATG GCT GCT AAA TTG CTT TTC TTC CTT TTG TTT CTG GTG TCT GCA CTA TCG GTC GCT TTA GCT GGT TTT GAG GAA GAT AAC CCT ATA AGG AGT GTT ACT CAA AGA CCA GAC AGC ATA GAA CCA GCT ATA TTG GGA GTT CTA GGT TCT TGC AGA CAT GCG TTT CAC TTC GCC AGA TTT GCT AGA AGA TAT GGT AAA TCG TAT GGT AGT GAA GAA GAG ATC AAG AAA CGT TTT GGG ATA TTT GTG GAA AAT TTG GCC TTC ATC AGG TCT ACT AAC AGA AAG GAC CTG AGC TAC ACA TTG GGG ATT AAT CAG TTT GCC GAC TTG ACT TGG GAG GAA TTT AGA ACC AAT CGT CTA GGT GCA GCA CAA AAT TGC TCT GCG ACT GCA CAT GGA AAC CAC AGA TTT GTG GAT GGA GTG TTA CCT GTT ACT AGA GAT TGG AGA GAA CAG GGT ATC GTT TCT CCC GTC AAA GAT CAA GGT TCA TGT GGC TCA TGT TGG ACG TTC TCT ACA ACA GGA GCC TTA GAA GCT GCC TAT ACC CAA TTG ACA GGG AAA TCC ACA AGT CTA AGC GAA CAA CAA CTG GTT GAT TGT GCT TCC GCA TTT AAC AAC TTT GGC TGT AAT GGT GGT TTA CCA AGT CAA GCT TTT GAA TAT GTC AAG TAT AAT GGA GGT ATT GAT ACA GAA CAA ACG TAT CCG TAT TTA GGC GTA AAC GGC ATT

TGC AAT TTC AAA CAG GAA AAT GTT GGG GTT AAA GTG ATT GAC TCC ATT AAC ATC ACG TTG GGT GCA GAG GAC GAG TTA AAA CAT GCT GTG GGT TTA GTT AGG CCT GTT TCA GTT GCC TTC GAA GTA GTC AAA GGT TTC AAC CTT TAC AAG AAA GGC GTT TAC TCT TCC GAC ACT TGT GGA AGA GAT CCA ATG GAT GTT AAT CAT GCA GTC TTG GCA GTA GGT TAC GGT GTA GAA GAT GGC ATT CCC TAT TGG CTT ATT AAG AAT TCA TGG GGT ACC AAT TGG GGC GAT AAC GGT TAC TTC AAG ATG GAG TTA GGA AAG AAT ATG TGC GGA GTA GCT ACC TGT GCC TCA TAC CCA ATT GTA GCG GTC TAA

c) VpHvVAN codon optimized for barley expression

ATG GCA GCT AAG CTC CTC TTC TTC CTG CTG TTT CTA GTG TCC GCC TTG TCC GTA GCG CTT GCT GGT TTC GAG GAG GAC AAC CCG ATT CGG TCG GTT ACC CAG AGG CCG GAT AGC ATT GAG CCT GCC ATC CTC GGT GTT CTA GGG TCA TGT CGG CAT GCC TTT CAC TTC GCT CGC TTT GCT CGC CGT TAT GGG AAG AGC TAT GGC TCC GAG GAA GAG ATC AAG AAG CGC TTC GGT ATC TTC GTA GAG AAC CTC GCC TTC ATT CGC TCG ACC AAT AGG AAG GAC CTT TCC TAC ACT CTC GGC ATC AAT CAG TTT GCC GAC CTC ACT TGG GAA GAG TTC AGG ACG AAC CGC TTG GGA GCC GCA CAA AAC TGC AGC GCT ACA GCG CAT GGC AAC CAC CGG TTC GTG GAT GGA GTT CTG CCT GTG ACA CGT GAT TGG AGA GAG CAA GGC ATC GTG TCT CCG GTT AAG GAT CAG GGA TCT TGC GGG TCT TGC TGG ACC TTT AGC ACG ACT GGG GCT CTG GAA GCC GCG TAC ACC CAA CTC ACA GGT AAA TCC ACG AGT CTC AGC GAA CAG CAA CTC GTC GAC TGT GCC AGT GCG TTC AAC AAT TTC GGC TGC AAT GGT GGG CTG CCA AGT CAG GCG TTC GAG TAC GTC AAG TAC AAT GGT GGA ATA GAC ACC GAA CAG ACC TAC CCC TAT CTC GGC GTC AAT GGC ATC TGC AAC TTC AAA CAG GAG AAC GTG GGC GTC AAG GTC ATA GAC TCC ATC AAC ATA ACG CTC GGA GCC GAG GAT GAG CTG AAG CAC GCA GTG GGC TTG GTC AGA CCA GTG TCA GTT GCA TTC GAG GTC GTG AAG GGC TTT AAC CTC TAC AAG AAA GGG GTG TAC TCG AGC GAT ACC TGT GGC AGG GAC CCA ATG GAC GTC AAT CAC GCG GTC CTT GCA GTG GGT TAT GGC GTC GAG GAT GGC ATC CCC TAC TGG CTG ATC AAG AAC TCT TGG GGC ACC AAC TGG GGT GAC AAC GGG TAC TTC AAG ATG GAG CTT GGC AAG AAC ATG TGC GGA GTA GCC ACA TGC GCG TCA TAC CCC ATT GTG GCT GTT TGA

d) Nicotiana benthamiana, cysteine proteinase

ATG TCT CGT TTC TCA CTC CTA TTG GCT CTC GTC GTC GCC GGT GGC CTT TTC GCC TCC GCA CTC GCC GGA CCG GCG ACC TTT GCC GAT GAG AAT CCG ATC AGA CAA GTC GTT TCT GAC GGT TTA CAT GAG CTG GAG AAC GCA ATT CTC CAA GTC GTC GGC AAG ACC CGC CAT GCT CTC TCC TTC GCT CGC TTT GCT CAC AGG TAT GGG AAG AGG TAC GAG TCA GTT GAG GAG ATA AAG CAA AGG TTC GAG GTA TTT TTG GAC AAT TTG AAG ATG ATT CGA TCG CAC AAC AAG AAA GGA CTA TCA TAC AAA CTC GGT GTC AAT GAG TTT ACC GAC CTA ACA TGG GAC GAG TTC CGG AGA GAC AGG TTG GGG GCA GCT CAA

AAC TGT TCA GCC ACC ACA AAG GGC AAT CTC AAA GTC ACT AAC GTT GTT CTG CCG GAG ACG AAA GAC TGG CGG GAA GCT GGG ATT GTC AGC CCA GTC AAG AAC CAG GGC AAG TGC GGA TCT TGC TGG ACA TTC AGC ACT ACT GGT GCA CTA GAA GCA GCA TAT AGC CAA GCA TTT GGG AAG GGA ATC TCT CTA TCT GAG CAG CAG CTT GTG GAC TGT GCT GGA GCT TTT AAT AAC TTT GGC TGC AAT GGT GGG CTC CCA TCA CAA GCC TTT GAG TAT ATT AAA TCC AAT GGT GGT CTT GAC ACT GAA GAA GCA TAT CCA TAC ACT GGC AAG AAT GGC TTA TGT AAA TTC TCA TCA GAA AAT GTT GGT GTC AAA GTC ATC GAT TCC GTC AAT ATT ACC CTG GGT GCT GAA GAT GAA CTA AAA TAC GCG GTT GCA TTG GTT AGG CCC GTT AGT ATA GCT TTT GAG GTG ATA AAA GGT TTC AAA CAA TAC AAG AGT GGT GTT TAC ACC AGC ACC GAA TGC GGC AAC ACT CCC ATG GAT GTA AAC CAT GCT GTT CTT GCT GTG GGT TAC GGT GTT GAA AAT GGT GTT CCC TAT TGG CTC ATC AAG AAT TCA TGG GGA GCA GAT TGG G

e) vpnbΔspΔ137van

ATG TCT CGT TTC TCA CTC CTA TTG GCT CTC GTC GTC GCC GGT GGC CTT TTC GCC TCC GCA CTC GGT TTC GAA GAA GAC AAT CCA ATC CGG TCC GTT ACA CAA AGG CCT GAC TCG ATT GAG CCT GCC ATC CTC GGC GTC CTT GGC AGT TGC CGC CAC GCC TTC CAC TTC GCA CGG TTC GCC CGC AGG TAC GGG AAG AGC TAC GGA TCG GAG GAG GAG ATC AAG AAG AGG TTC GGG ATC TTC GTG GAG AAT CTA GCG TTT ATC CGG TCC ACT AAT CGG AAG GAT CTG TCG TAT ACC CTA GGA ATC AAC CAA TTC GCC GAC CTG ACC TGG GAG GAA TTC CGG ACC AAT CGC CTT GGT GCG GCG CAG AAC TGC TCG GCG ACT GCG CAT GGA AAC CAC CGG TTT GTC AAC GTT GTT CTG CCG GAG ACG AGG GAT TGG AGG GAG CAA GGG ATA GTG AGC CCT GTA AAG GAC CAA GGA AGC TGT GGA TCT TGC TGG ACT TTC AGT ACT ACT GGA GCA CTA GAG GCT GCA TAT ACA CAG CTA ACT GGA AAG AGC ACA TCA TTA TCT GAA CAG CAA CTT GTG GAC TGT GCC TCA GCA TTC AAT AAC TTT GGA TGC AAT GGA GGT TTG CCT TCC CAA GCC TTT GAA TAC GTT AAG TAC AAT GGA GGC ATC GAC ACA GAA CAG ACT TAT CCA TAC CTT GGT GTC AAT GGT ATC TGC AAC TTC AAG CAG GAG AAT GTT GGT GTC AAG GTC ATT GAT TCG ATA AAC ATC ACC CTG GGT GCT GAG GAT GAG TTG AAG CAT GCA GTG GGC TTG GTG CGT CCA GTT AGC GTT GCA TTT GAG GTT GTG AAA GGT TTC AAT CTG TAC AAG AAA GGT GTA TAC AGC AGT GAC ACC TGT GGA AGA GAT CCA ATG GAT GTG AAC CAC GCA GTT CTT GCC GTC GGT TAT GGA GTC GAG GAC GGG ATT CCT TAT TGG CTC ATC AAG AAC TCA TGG GGT ACA AAT TGG GGT GAC AAT GGC TAC TTT AAG ATG GAA CTC GGC AAG AAC ATG TGT GGT GTT GCA ACT TGC GCA TCT TAT CCC ATT GTG GCT GTG TAG

f) GhVAN

ATG GCT CGC CTT CTG CTG CTC CTC GTC GGA GTT CTG ATC GCC TGC GCC GCC GGC GCA AGA GCC GGA TCG GAG TTC CTC GCC GAG GAT AAT CCG ATC AGG CAA GTC GTC GAC GGT ATG CAC GAA CTC GAG TCG TCT ATT CTC AAA GCA GTC GGC AAC TCG CGC CGC GCC TTC TCC TTC GCT CGC TTT GCT CAT AGA TAC GGG AAG AGC TAC GAG AGT TCG GAG GAG ATA CAG AAG AGG TTC CAA GTT TAC TCT GAG AAT TTG AGG ATG ATC CGA TCG CAT AAC AAG AAA GGA CTA TCC TAT TCC ATG GGC GTT AAC GAG TTC TCT GAT CTG ACA TGG GAC GAG TTC AAA AAG CAT AGA TTG GGA GCT GCT CAA AAT TGC TCC GCT ACA AGA AGG GGC AAT CAT AAG CTC ACC AGT GCT ATC CTT CCG GAC TCG AAA GAC TGG AGG GAA AGT GGC ATT GTT AGC CCA GTG AAA AGT CAA GGT AGC TGT GGA TCT TGC TGG ACA TTC AGT TCA ACT GGA GCA CTG GAG GCA GCT TAT GCA CAA GCA TTC GGA AAG GGT ATT TCT CTG TCT GAG CAG CAG CTC GTT GAT TGT GCT GGA GCT TTC AAC AAC TTT GGC TGC AAT GGT GGA TTG CCC TCT CAA GCC TTC GAA TAC ATC AAA TAT AAC GGT GGT CTT ATG ACT GAG GAG GCA TAT CCA TAT ACT GGT CAT GAT GGA GAA TGC AAG TAT TCC TCT GAA AAT GCT GCC GTC CAA GTA CTT GAC TCT GTC AAT ATC ACC CTG GGT GCT GAA GAT GAA CTT AAG CAC GCA GTT GCA TTG GTT CGG CCA GTA AGT GTG GCA TTT GAG GTT GTT GAT GGA TTC CGA TCA TAC AAT GGT GGA GTT TAC ACT AGC ACT ACT TGT GGC AGC GAT CCA ATG GAT GTA AAC CAT GCT GTT CTT GCT GTT GGT TAC GGA GTT GAA GGT GGC GTG CCG TAC TGG CTA ATC AAG AAT TCA TGG GGA GCT GAC TGG GGG GAC CAA GGC TAC TTC AAA ATG GAG ATG GGC AAG AAC ATG TGT GGT GTT GCA ACA TGT GCA TCA TAC CCT GTA GTT GCT TAA

*g) Vp*VAN amino acid sequence

MAAKLLFFLLFLVSALSVALAGFEEDNPIRSVTQRPDSIEPAILGVLGSCRHAFHFARFARRYGKSYGSEEEIKKRFGIFVE
NLAFIRSTNRKDLSYTLGINQFADLTWEEFRTNRLGAAQNCSATAHGNHRFVDGVLPVTRDWREQGIVSPVKDQGSC
GSCWTFSTTGALEAAYTQLTGKSTSLSEQQLVDCASAFNNFGCNGGLPSQAFEYVKYNGGIDTEQTYPYLGVNGICNFK
QENVGVKVIDSINITLGAEDELKHAVGLVRPVSVAFEVVKGFNLYKKGVYSSDTCGRDPMDVNHAVLAVGYGVEDGIPY
WLIKNSWGTNWGDNGYFKMELGKNMCGVATCASYPIVAV

▼:Putative protease cleavage site

*: Amino acid residues involved in stabilizing the oxyanion hole

Amino acid residues in bold: A putative ER-targeting signal peptide

Amino acid residues in blue: Conserved residues known to contribute to the formation of the active site in cysteine proteinases

Amino acids with wavy underline: GCxGG domain found in papaine-like cysteine proteinases

Amino acid residues in gray: Conserved cysteins involved in disulfide bridge formation in cysteine proteinases

Underlined amino acid residues: ERFNIN motif

Boxed amino acids: Putative sumoylation site

Supplementary Figure 3: Enzyme activity of *Vp*UGT72U1 and *At*UGT72E2 with ferulic acid

Yeast strains integrated with *Vp*UGT72U1 and *At*UGT72E2 were grown on synthetic complete (SC) medium supplemented with 2.5mM of ferulic acid and after 72h cells were harvested for LC-metabolic profiling.

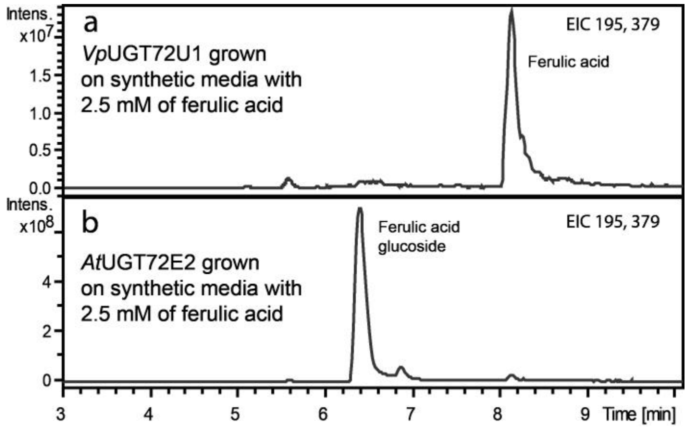

(a) *Vp*UGT72U1 stably integrated yeast was grown on SC medium supplemented with 2.5mM of ferulic acid EIC 195: Extracted ion chromatogram m/z (ferulic acid mw + H^+) and EIC 379: Extracted ion chromatogram m/z (ferulic acid glucoside mw + Na^+)

(b) AtUGT72E2 stably integrated yeast were grown on synthetic media with 2.5mM of ferulic acid. EIC 195: Extracted ion chromatogram m/z (ferulic acid mw + H^+) and EIC 379: Extracted ion chromatogram m/z (ferulic acid glucoside mw + Na^+)

*Vp*UGT72U1 is not able to glycosylate ferulic acid (a) while *At*UGT72E2 is very efficiently glycosylate ferulic acid to ferulic acid glucoside (b).

Supplementary Figure 4: Substrate specificities of *VpScVAN* and *vpscΔspvan* were assayed in *S. cerevisiae*.

Yeast harbouring stably integrated *VpVAN*, *VpScVAN* codon optimized for yeast expression, truncated *VpVAN* devoid of the signal peptide (*vp Δsp van*)or truncated *VpScVAN* codon optimized and devoid of the signal peptide (*vpscΔsp van*) were tested in combination with a stably integrated *AtUGT72E2*. The strains were incubated with ferulic acid for 72 h in synthetic complete medium before metabolite profiles were determined by LC-MS.

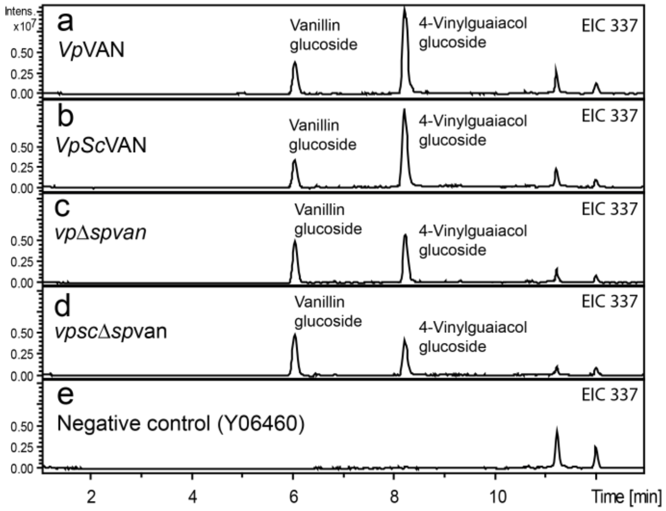

Formation of vanillin glucoside was observed with yeast fed with ferulic acid (RT 6 min) in all strains except the negative control where no vanillin or vanillin glucoside observed. Administrating yeast with ferulic acid resulted in production of aroma compound 4-vinylguaiacol glucoside in high concentrations due to yeast ferulate decarboxylase activity (RT 8.2 m). EIC 337: m/z Vanillin glucoside (M + Na+)

Supplementary Figure 5: The biological activity of vanillin synthase was also assessed by a stable expression in barley

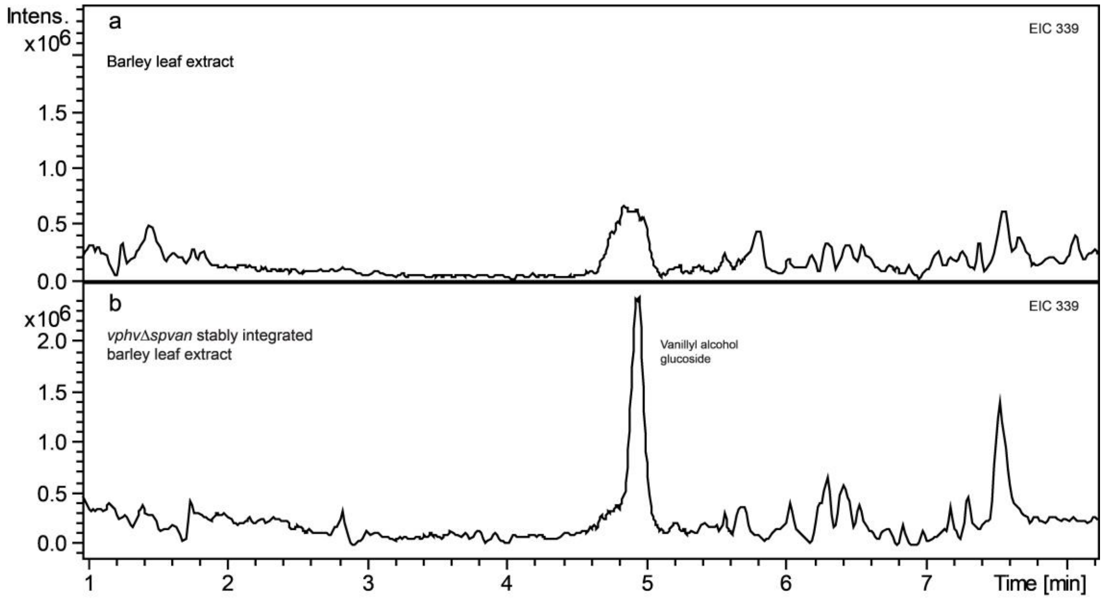

VpHvVAN gene sequence was modified to encode a D-hordein signal peptide as a replacement for the original vanilla ER-targeting signal peptide (*vphv*Δ*spva*n). A constitutive ubiquitin promoter was used to drive the expression of both genes. Leaf samples from successfully transformed plants were collected 6-8 weeks after transfer of plantlets to the greenhouse and metabolic profiling was carried out by LC-MS. Barley plants transformed with *vphvΔspvan* were found to accumulate vanillyl alcohol glucoside in higher levels than control plants (b) EIC 339: m/z vanillyl alcohol glucoside (M + Na+)

Supplementary Table 1: Plasmids constructed in this study

Plasmid	Backbone	Gene content	Plasmid type	Selection marker
pNG1	p426-GPD	*VpVAN* from *V. planifolia* made with PCR from cDNA lib with SpeI and BamHI sites	2 micron (*S. cerevisiae*)	URA3
PNG4	pBS II SK(+)	*VpScVAN* synthetically made from MWG for yeast expression XbaI and BamHI		
pNG5	p426-GPD	*OMT1 V. planifolia* made with PCR from cDNA lib	2 micron (*S. cerevisiae*)	URA3
pNG6	p426-GPD	*OMT1a V. planifolia* made with PCR from cDNA lib	2 micron (*S. cerevisiae*)	URA3
pNG7	p426-GPD	*OMT3 V. planifolia* made with PCR from cDNA lib	2 micron (*S. cerevisiae*)	URA3
pNG8	p426-GPD	*OMT3a V. planifolia* made with PCR from cDNA lib	2 micron (*S. cerevisiae*)	URA3
pNG9	p426-GPD	*OMT3b V. planifolia* made with PCR from cDNA lib	2 micron (*S. cerevisiae*)	URA3
pNG10	p426-GPD	*OMT3c V. planifolia* made with PCR from cDNA lib	2 micron (*S. cerevisiae*)	URA3
pNG11	p426-GPD	*OMT4 V. planifolia* made with PCR from cDNA lib	2 micron (*S. cerevisiae*)	URA3
pNG12	p426-GPD	*OMT5a V. planifolia* made with PCR from cDNA lib	2 micron (*S. cerevisiae*)	URA3
pNG13	p426-GPD	*OMT5b V. planifolia* made with PCR from cDNA lib	2 micron (*S. cerevisiae*)	URA3
pNG14	p426-GPD	*OMT5c V. planifolia* made with PCR from cDNA lib	2 micron (*S. cerevisiae*)	URA3
pNG15	P416TEF	*OMT5c V. planifolia* made with PCR from cDNA lib	*CEN*-ARS (*S. cerevisiae*)URA3	URA3
pNG16	P416TEF	*OMT1 V. planifolia* made with PCR from cDNA lib	*CEN*-ARS (*S. cerevisiae*)	URA3
pNG17	P416TEF	*OMT1a V. planifolia* made with PCR from	*CEN*-ARS (*S.*	URA3

		cDNA lib	*cerevisiae*)	
pNG18	P416TEF	*OMT3 V. planifolia* made with PCR from cDNA lib	*CEN*-ARS (*S. cerevisiae*)	URA3
pNG19	P416TEF	*OMT3a V. planifolia* made with PCR from cDNA lib	*CEN*-ARS (*S. cerevisiae*)	URA3
pNG20	P416TEF	*OMT3b V. planifolia* made with PCR from cDNA lib	*CEN*-ARS (*S. cerevisiae*)	URA3
pNG21	P416TEF	*OMT3c V. planifolia* made with PCR from cDNA lib	*CEN*-ARS (*S. cerevisiae*)	URA3
pNG22	P416TEF	*OMT4 V. planifolia* made with PCR from cDNA lib	*CEN*-ARS (*S. cerevisiae*)	URA3
pNG23	P416TEF	*OMT5a V. planifolia* made with PCR from cDNA lib	*CEN*-ARS (*S. cerevisiae*)	URA3
pNG24	P416TEF	*OMT5b V. planifolia* made with PCR from cDNA lib	*CEN*-ARS (*S. cerevisiae*)	URA3
pNG25	P416TEF	*OMT5c V. planifolia* made with PCR from cDNA lib	*CEN*-ARS (*S. cerevisiae*)	URA3
pNG26	topo blunt end	*CYP98A3* cloned from *V.planifolia* cDNA lib		
pNG27	PYES2-Des52	*VpVAN* with his tag gateway cloning from cDNA to des vector	Plant gateway vector	Kan, carb, Rif
pNG28	pVAN714	pTEF1-*VpVAN* + pPGK1-*AtUGT72E2*	Integration (*S. cerevisiae*)	URA3
pNG29	pVAN714	pTEF1-*VpScVAN* codon opt. + pPGK-*At*UGT72E2	Integration (*S. cerevisiae*)	URA3
pNG30	pVAN714	pTEF1-*VpVAN* codon opt.	Integration (*S. cerevisiae*)	URA3
pNG31	pVAN714	pTEF1-*Vp*VAN + pPGK1-*VpUGT72U1*	Integration (*S. cerevisiae*)	URA3
pNG32	pVAN714	pTEF1- *vpΔsp van* + pPGK1-*AtUGT72E2*	Integration (*S. cerevisiae*)	URA3
pNG33	pVAN714	pTEF1- *vpsc Δspvan* + pPGK1-*AtUGT72E2*	Integration (*S. cerevisiae*)	URA3

pNG34	pEAQ-HT-DEST3	*VpVAN* cloning from cDNA to des vector	Plant gateway destination vector	Kan
pNG27	PYES2-Des52	*VpVAN* with his tag gateway cloning from cDNA to des vector	Plant gateway vector	Kan, carb, Rif
pNG35	pEAQ-HT-DEST3	*vp Δ61 van*	Plant gateway destination vector	Kan
PNG36	pEAQ-HT-DEST3	*vp Δ137 van*	Plant gateway destination vector	Kan
pNG37	pEAQ-HT-DEST3	*vp nb Δsp Δ137 van*	Plant gateway destination vector	Kan
pNG38	pEAQ-HT-DEST3	*GhVAN*	Plant gateway destination vector	Kan
pNG39	pWBVec8-UbiUSERNOS	*VpHvVAN* codon optimized for barley expression	Barley stable gene expression vector	spectinomyci
pNG40	pWBVec8-Ubi::HorDSP-USERNOS	*vp hvΔsp van* codon optimized for barley expression	Barley stable gene expression vector	spectinomyci

Supplementary Table 2: Yeast strains constructed in this study

Species	strain name	Relevant genotype
Saccharomyces cerevisiae	Y06460:: *Vp*VAN:: *At*UGT72E2	MATa his3-D1 leu2-D0 met 15-d0 ura3-D0 YMR318C (adh6)::KanMX4 (exg1-d0)::(pNG28)::URA3
Saccharomyces cerevisiae	Y06460:: *VpSc*VAN codon opt.:: *At*UGT72E2	MATa his3-D1 leu2-D0 met 15-d0 ura3-D0 YMR318C (adh6)::KanMX4 (exg1-d0)::(pNG29)::URA3
Saccharomyces cerevisiae	Y06460:: *VpSc*VAN	MATa his3-D1 leu2-D0 met 15-d0 ura3-D0 YMR318C (adh6)::KanMX4 (exg1-d0)::(pNG31)::URA3
Saccharomyces cerevisiae	Y06460:: *VpSc*VAN codon opt.:: *Vp*UGT72U1	MATa his3-D1 leu2-D0 met 15-d0 ura3-D0 YMR318C (adh6)::KanMX4 (exg1-d0)::(pNG30)::URA3
Saccharomyces cerevisiae	Y06460:: *Vp*ΔSP VAN:: *At*UGT72E2	MATa his3-D1 leu2-D0 met 15-d0 ura3-D0 YMR318C (adh6)::KanMX4 (exg1-d0)::(pNG32)::URA3
Saccharomyces cerevisiae	Y06460:: *VpSc*ΔSPVAN codon opt.:: *At*UGT72E2	MATa his3-D1 leu2-D0 met 15-d0 ura3-D0 YMR318C (adh6)::KanMX4 (exg1-d0)::(pNG33)::URA3

Supplementary Table 3: Primers used in this study

Candidate genes	Oligo nucleotide used for cloning
pYes2 Forward	ATG CAA AAA CTG CAT AAC C
pYes2 Reverse	TAG ATG CAT GCT CGA GCG G
*Vp*VAN Forward	ATT TAC TAG TAA AAT GGC AGC TAA GCT C
*Vp*VAN Reverse	ATT TAG GAT CCC TAC ACA GCC ACA ATG
*In situ Vp*VAN Forward	AAG CCT TTG AAT ACG TTA AGT ACA ATG GA
*In situ Vp*VAN Reverse	GTG TCA CTG CTG TAT ACA CCT TTC TT
TNT *Vp*VAN Forward	GAT CCT AAT ACG ACT CAC TAT AGG GAA CAG CCA CCA TGG CAG CTA AGC TCC TCT T
TNT *Vp*VAN Reverse	TTT TTT TTT TTT TTT TTT TTT TTT ACT ACA CAG CCA CAA TGG GAT AAG ATG CGC AAG TTG C
*Vp*VAN gateway Forward	GGG GAC AAG TTT GTA CAA AAA AGC AGG CTA AAA ATG TCT ATG GCA GCT AAG CTC CTC TTC
*Vp*VAN gateway Reverse	GGG GAC CCA GCT TTC TTG TAC AAA GTG GTC ACA GCC ACA ATG GGA TAA GAT G
*VpHv*VAN forward	GGTCTTAAUATGGCAGCTAAGCTCCTC
*VpHv*VAN reverse	GGCATTAAU TCAAACAGCCACAATGGGGTATG
*VpHv*ΔSPVAN forward	GGTCTTAAUATGTTCCTGCTGTTTCTAGTGTCCG

TNT *Vp*Δ61VAN forward	GGATCCTAATACGACTCACTATAGGGAACAGCCACC ATG AGATATGGTAAATCGTATGGTAGTG
TNT *Vp*Δ61VAN reverse	TTTTTTTTTTTTTTTTTTTTTTTTTTTTTTTTTTAGACCGCTACAATTGGG TATG
*Vp*Δ61VAN gateway forward	GGG GAC AAG TTT GTA CAA AAA AGC AGG CTT CAA AA ATG TCTT CGA TGA GGT ACG GGA AGA GCT ACG GAT CGG AG
*Vp*Δ61VAN gateway reverse	GGG GAC CAC TTT GTA CAA GAA AGC TGG GT CTACACAGCCACAATGGGATAAG
TNT *Vp*Δ137VAN forward	GGATCCTAATACGACTCACTATAGGGAACAGCCACC ATG TTACCTGTTACTAGAGATTGGAG
TNT *Vp*Δ137VAN reverse	TTTTTTTTTTTTTTTTTTTTTTTTTTTTTTTTTTAGACCGCTACAATTGGG TATG
*Vp*Δ137VAN gateway forward	GGGG ACA AGT TTG TAC AAA AAA GCA GGC TTC AAAA ATG TCT TCG ATG GCG TGC TTC CTG TAA CGA GGG A
*Vp*Δ137VAN gateway reverse	GGG GAC CAC TTT GTA CAA GAA AGC TGG GT CTACACAGCCACAATGGGATAAG
*Gh*VAN gateway forward	GGG GAC AAGT TTG TAC AAA AAA GCA GGC T TC AAAA ATGGCTCGCCTTCTGCTGCTC
*Gh*VAN gateway reverse	GGG GAC CAC TTT GTA CAA GAA AGC TGG GT TTAAGCAACTACAGGGTATGATG
*Vp*UGT72U1 Forward	ATCAACGGGUAAAATGGAGAGCAATCCCAACCG
*Vp*UGT72U1 Reverse	CGTGCGAUTCACTTGCCACTGAGAACCG
